# BILFF: All-Atom Force Field for Modeling Triazolium- and Benzoate-Based Ionic Liquids

**DOI:** 10.3390/molecules28227592

**Published:** 2023-11-14

**Authors:** Eliane Roos, Daniel Sebastiani, Martin Brehm

**Affiliations:** 1 Institut für Chemie—Theoretische Chemie, Martin-Luther-Universität Halle–Wittenberg, Von-Danckelmann-Platz 4, 06120 Halle (Saale), Germany; eliane.roos@gmx.de (E.R.); daniel.sebastiani@chemie.uni-halle.de (D.S.); 2 Department Chemie, Universität Paderborn, Warburger Straße 100, 33098 Paderborn, Germany

**Keywords:** ionic liquid, cellulose solvent, force field molecular dynamics simulation, solubility, hydrogen bond, solvation shell, imidazolium, triazolium, benzoate, acetate

## Abstract

We present an extension of our previously developed all-atom force field BILFF (Bio-polymers in Ionic Liquids Force Field) to three different ionic liquids: 1-ethyl-3-methyl-1,2,3-triazolium acetate ([EMTr][OAc]), 1-ethyl-3-methyl-1,2,3-triazolium benzoate ([EMTr][OBz]), and 1-ethyl-3-methylimidazolium benzoate ([EMIm][OBz]). These ionic liquids are of practical importance as they have the ability to dissolve significant amounts of cellulose even at room temperature. Our force field is optimized to accurately reproduce the strong hydrogen bonding in the system with nearly quantum chemical accuracy. A very good agreement between the microstructure of the quantum chemical simulations over a wide temperature range and experimental density data with the results of BILFF were observed. Non-trivial effects, such as the solvation shell structure and π–π stacking of the cations, are also accurately reproduced. Our force field enables accurate simulations of larger systems, such as solvated cellulose in different (aqueous) ionic liquids, and is the first to present the optimized parameters for mixtures of these solvents and water.

## 1. Introduction

Ionic liquids (ILs) have become an attractive alternative to conventional solvents in various industries due to their unique properties like their non-volatility, non-flammability, and their very good thermal and chemical stability [1,2]. The properties of ILs can be tailored to suit specific applications by changing the cation or anion components [3,4]. This flexibility has led to their use in a range of different applications, including in catalysis, electrochemistry, material science, energy, and biotechnology [5,6,7,8,9,10,11]. One of the promising usage of ILs is the dissolution and processing of cellulose [12,13,14,15,16,17], which enables the production of cellulose-based derivatives in a homogeneous manner [18,19] and the processing of biomass by the separation of wood components [20,21].

Cellulose is characterized by its highly structured composition of glucose monomers, forming long chains that can range from several hundred to tens of thousands of units in length. These chains are highly ordered and arranged in parallel. Intramolecular hydrogen bonding stiffens the polymer chains, while intermolecular hydrogen bonding facilitates the arrangement of these linear polymers into sheet-like structures. These sheets are densely packed together through hydrophobic interactions and form crystalline structures [22,23]. To dissolve cellulose in ILs, the intermolecular hydrogen bonds between the cellulose strands must be disrupted and replaced by interactions with the solvent. This can be achieved through the formation of new hydrogen bonds to the solvent molecules. Several studies have demonstrated that the effectiveness of ILs in dissolving cellulose depends on several factors such as the type of cation and anion, the basicity as well as the position and length of the alkyl chains of the cation [15,16,17,24,25]. Therefore, the choice of the anion plays a more decisive role than the choice of the cation; the IL 1-ethyl-3-methylimidazolium acetate ([EMIm][OAc]), for example, shows one of the highest cellulose solubilities with 36.0 g per mol IL, while 1-ethyl-3-methyl-1,2,3-triazolium acetate ([EMTr][OAc]) has a barely reduced solubility of 34.0 g per mol IL. In contrast, the ILs 1-ethyl-3-methyl-1,2,3-triazolium benzoate ([EMTr][OBz]) and 1-ethyl-3-methylimidazolium benzoate ([EMIm][OBz]) have decreased cellulose solubilities of 21.7 g per mol IL and 18.6 g per mol IL, respectively [15,16].

The two cations, [EMTr]${}^{+}$ and [EMIm]${}^{+}$, are distinguished by the replacement of one ring carbon atom in [EMTr]${}^{+}$ by a third nitrogen atom. As a result, the third ring proton in the 1,2,3-triazolium is missing. This structural change leads to a lower probability of proton abstraction and thus to a lower formation of *N*-heterocyclic carbenes (NHC). NHCs are highly reactive and form dimers or undesirable byproducts. Thus, they can also cause the unwanted decomposition of cellulose [26]. The potential use of triazolium-containing ILs as non-derivatizing cellulose solvents is nearly unexplored and was first described by Brehm et al. in 2019 [15]. By understanding the complex mechanisms involved in cellulose dissolution in different ILs, it is possible to develop more efficient and sustainable processes for the production of cellulose-based materials. This requires a thorough understanding of the interactions between ILs and cellulose, which can be achieved through molecular dynamics (MD) simulations. However, the complexity of the system, together with the problem that solubility processes take place on time scales that exhaust the resources of quantum chemical MD simulations, necessitates the use of force field MD (FFMD) simulations. To accurately simulate the interactions between ILs and cellulose, it is crucial to have well-optimized force field parameters for the individual ions. Until now, there has been no optimized force field available for the ions [EMTr]${}^{+}$ and [OBz]${}^{-}$, which limited the accuracy of simulations involving these ILs. In this article, we present the development of a force field for (aqueous) [EMTr][OAc], [EMIm][OBz] and their combination [EMTr][OBz], which is an extension of our previously published force field for [EMIm]${}^{+}$ and [OAc]${}^{-}$ called BILFF (Bio-Polymers in Ionic Liquids Force Field) [27,28]. The force field development is focused on the accurate reproduction of the microstructure of the ILs, especially the hydrogen bonds, based on results of ab initio MD (AIMD) simulations. To optimize the force field parameters, we compared the results of different analysis like radial and combined distribution functions of the hydrogen bonds along with statistical analyses of bond lengths, angles, and dihedral angles with the results from AIMD simulations for both the pure and aqueous ILs. Based on this comparison, the force field parameters were adjusted and optimized for a simulation of the ILs both in the absence and presence of water at 350 K. For the aqueous system, a ratio of IL ion pairs to water of 1:3 was chosen, at which cellulose is already precipitated again [29,30,31].

The results demonstrate that the new force field effectively reproduces the structural and dynamic properties of the ILs, including ion pairing, solvation shell structure, and hydrogen bonding with respect to their geometry and lifetime, over a wide temperature range. Furthermore, we contrasted and compared the results of the three ILs [EMTr][OAc], [EMIm][OBz] and [EMTr][OBz] with experimental density data, as well as with the previously published results for [EMIm][OAc].

The optimized force field parameters enable the simulation of larger cellulose systems in various ILs on larger time scales, which would otherwise be too computationally expensive when using AIMD simulations. This enables a deeper understanding of the underlying interactions for the solubility of cellulose in the different ILs and thus the identification of novel and improved cellulose solvents.

## 2. Optimization Procedure

**Figure 1:**
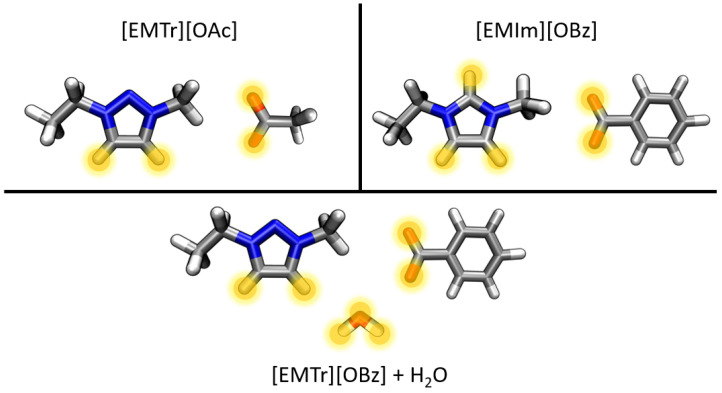
Overview of the investigated systems containing the ions 1-ethyl-3-methyl-1,2,3-triazolium ([EMTr]${}^{+}$), 1-ethyl-3-methylimidazolium ([EMIm]${}^{+}$), benzoate ([OBz]${}^{-}$), and acetate ([OAc]${}^{-}$) in this article with highlighted relevant hydrogen bonds donors and acceptors (atom color code: blue—N; grey—C; red—O; white—H).

In a previously published article [27], the optimized force field parameters for (aqueous) [EMIm][OAc] were presented. The aim of this article is the extension of the parameter set to the ions [EMTr]${}^{+}$ and [OBz]${}^{-}$ using the already optimized parameter set for [OAc]${}^{-}$ and [EMIm]${}^{+}$. For this purpose, simulations of [EMTr][OAc] and [EMIm][OBz] were performed. Since it is known that many imidazolium- and triazolium-based ionic liquids are hygroscopic and that this might drastically affect their properties [32,33], the optimization of the force field parameters was carried out simultaneously in the presence and absence of water. For this purpose, simulations of pure [EMIm][OBz] and [EMTr][OAc] as well as the combination of both ions as [EMTr][OBz] in water, were performed (cf. Figure 1). The latter simulation is used to investigate both parameter sets of [EMIm][OBz] and [EMTr][OAc] in the presence of water. The optimization of the force field parameters was performed by comparing various analyses of force field MD simulations with reference AIMD simulations of these three systems, which are discussed in more detail in the following sections.

The aim of optimizing the force field parameters for [EMTr]${}^{+}$ and [OBz]${}^{-}$ in the presence and absence of water is to enable force field MD simulations of these ions with nearly the accuracy of quantum chemical calculations to compute larger systems with less computational effort.

The focus was set on the correct reproduction of the hydrogen bonds between the investigated ions [EMTr]${}^{+}$ as well as [OBz]${}^{-}$ and their counterions [OAc]${}^{-}$ and [EMIm]${}^{+}$ calculated from quantum chemical simulations. [EMIm]${}^{+}$ and [OAc]${}^{-}$ were chosen as counterions for [OBz]${}^{-}$ and [EMTr]${}^{+}$, since optimized force field parameters are already available for these two ions (see our previously published articles on BILFF [27,28]).

The hydrogen bonds in the aqueous system were investigated using an AIMD simulation of aqueous [EMTr][OBz]. A molar fraction of $\chi_{\mathrm{IL}}=0.25$ was simulated in order to cover a wide range of applications as it is known that, at this water content, cellulose is already precipitated again in other ILs such as [EMIm][OAc] [29,30,31].

The force field is based on the functional form of the OPLS–AA force field [34,35,36]:

(1) $$\begin{array}{c} \begin{array}{cc} U(r^{N})= & \sum_{i\;\in \;\mathrm{bonds}}k_{l,i}{\left(l_{i}-l_{i,0}\right)}^{2}+\sum_{i\;\in \;\mathrm{angles}}k_{\theta ,i}{\left(\theta_{i}-\theta_{i,0}\right)}^{2}\\ \; & +\sum_{i\;\in \;\mathrm{dihedrals}}[\frac{V_{i,1}}{2}\left[1+\cos\left(\phi_{i}\right)\right]+\frac{V_{i,2}}{2}\left[1-\cos\left(2\phi_{i}\right)\right]\\ \; & \;+\frac{V_{i,3}}{2}\left[1+\cos\left(3\phi_{i}\right)\right]+\frac{V_{i,4}}{2}\left[1-\cos\left(4\phi_{i}\right)\right]]\\ \; & +\sum_{i=1}^{N}\sum_{j=i+1}^{N}\left[4\epsilon_{ij}\left[{\left(\frac{\sigma_{ij}}{r_{ij}}\right)}^{12}-{\left(\frac{\sigma_{ij}}{r_{ij}}\right)}^{6}\right]+\frac{q_{i}q_{j}e^{2}}{4\pi \epsilon_{0}r_{ij}}\right]f_{ij} \end{array} \end{array}$$

(2) $$\begin{array}{c} \epsilon_{i,j}=\sqrt{\epsilon_{i}\;\epsilon_{j}},\;\sigma_{i,j}=\sqrt{\sigma_{i}\;\sigma_{j}}\;. \end{array}$$

In order to optimize the force field parameters, radial distribution functions of the reference AIMD simulations were compared with the results of the force field MD simulations. To retain the properties of the underlying force fields as much as possible, the force field parameters *q* and σ were iteratively adjusted using a trial-and-error approach. The force field parameters were varied until the deviations of the RDFs were minimized. The results are discussed in Section 2.1.1. In addition, σ was optimized with respect to a good reproduction of the experimental density (see Table 7).

**Figure 2:**
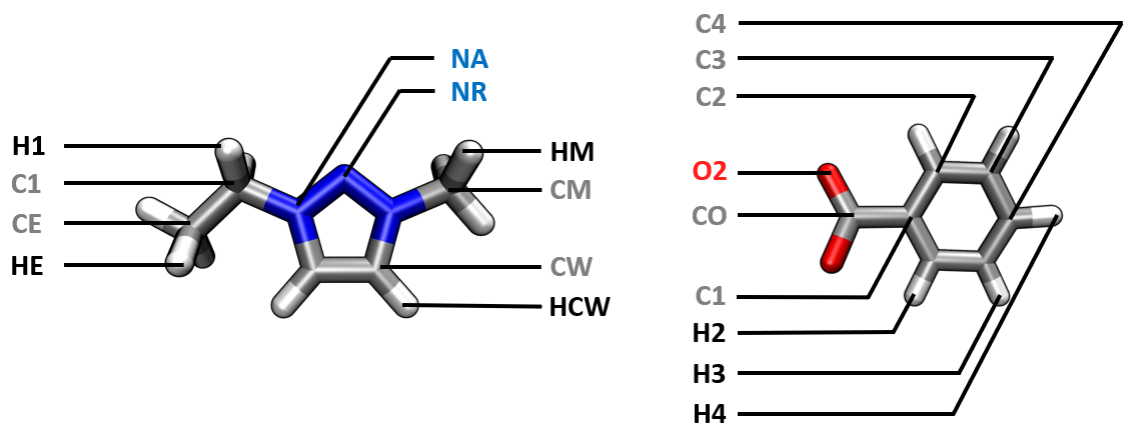
Nomenclature of the atom types of [EMTr]${}^{+}$ and [OBz]${}^{-}$ in our force field BILFF.

The bonded interactions were adjusted based on a comparison of the statistical occurrence of bond lengths, bond angles and dihedral angles, and by varying the force constants and height of the potential barrier. The nomenclature of the atom types can be found in Figure 2.

To correctly reproduce the reference AIMD simulations, the equilibrium bond length of the NA–NR bond in [EMTr]${}^{+}$, for example, was modified by +0.3%. The NA–CW bond length was reduced by about 2%, while the bond force constant was increased by about 12% compared to the value in [EMIm]${}^{+}$.

In [OBz]${}^{-}$, the greatest adjustment in the bond length and force constant occurred in the CO–O2 bond, with changes of about +2% and +56%, respectively. The equilibrium bond angle, on the other hand, was not changed for both ions.

**Figure 3:**
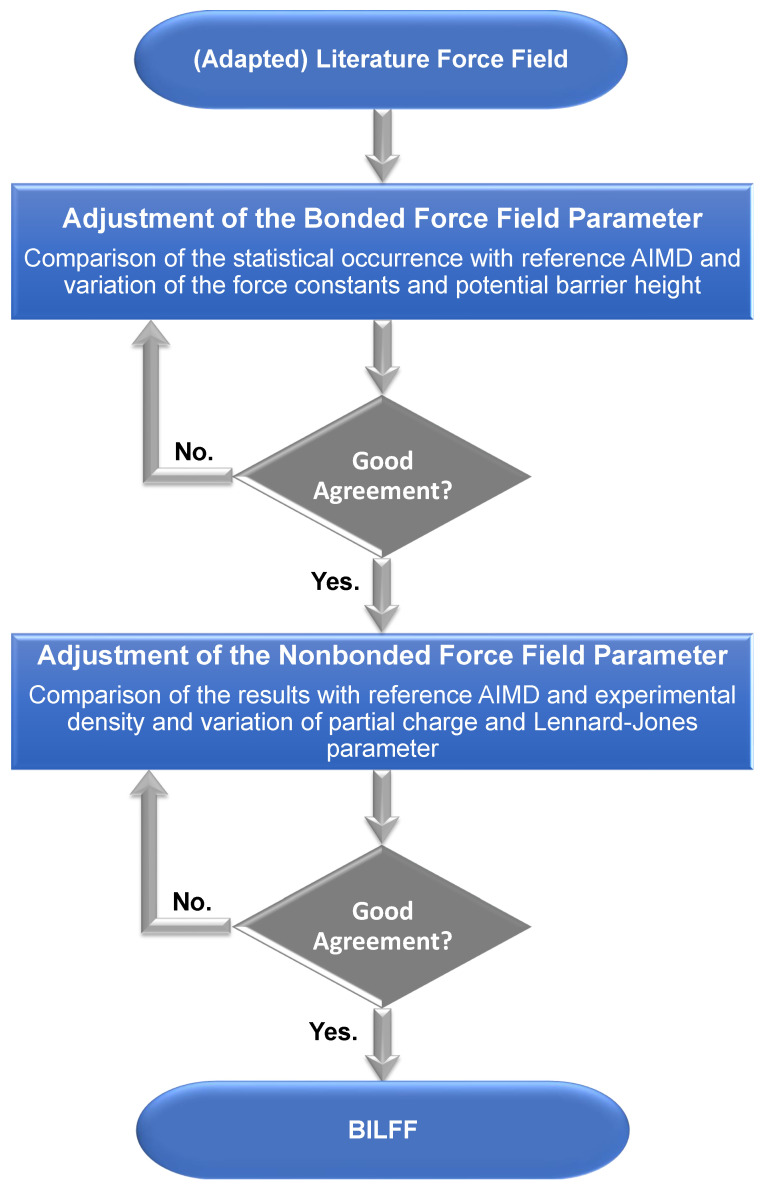
Flowchart of the optimization process of the force field parameters for BILFF. As a starting point for the optimization of the force field parameters, BILFF [27,28] for [EMTr]${}^{+}$ was used. For [OBz]${}^{-}$ the Lennard-Jones parameters and force constants were adapted from OPLS–AA [34,35,36]. The partial charges were calculated using the restrained electrostatic potential (RESP) methodology. The equilibrium bond length was calculated using a geometry optimization.

A flowchart of the force field optimization process can be found in Figure 3. The force field parameters and nomenclature for the atom types and classes can be found in Table 1, Table 2, Table 3, Table 4 and Table 5.

### 2.1. Microstructure of the Systems

#### 2.1.1. Radial Distribution Functions

**Figure 4:**
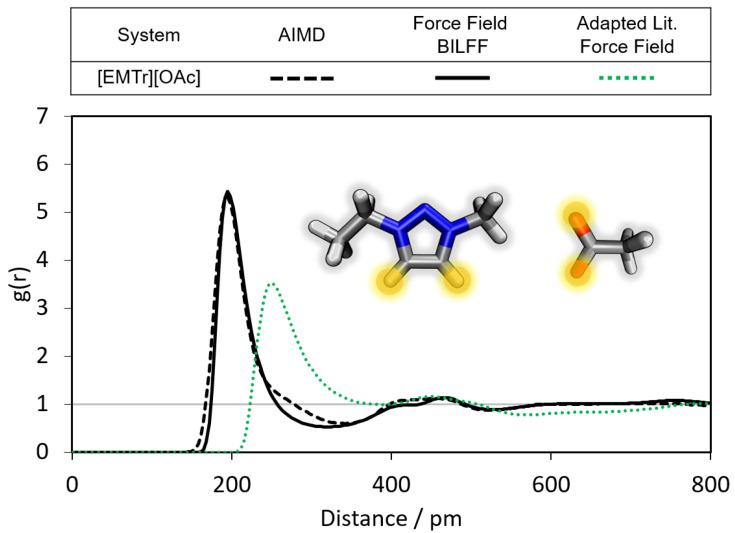
Comparison of the RDFs of the hydrogen bond between the marked oxygen atoms of [OAc]${}^{-}$ and the ring protons of [EMTr]${}^{+}$ calculated from a reference AIMD and force field MD simulations using adapted literature force field parameters [37,38,39] and BILFF. The RDFs are averaged over both ring protons.

**Figure 5:**
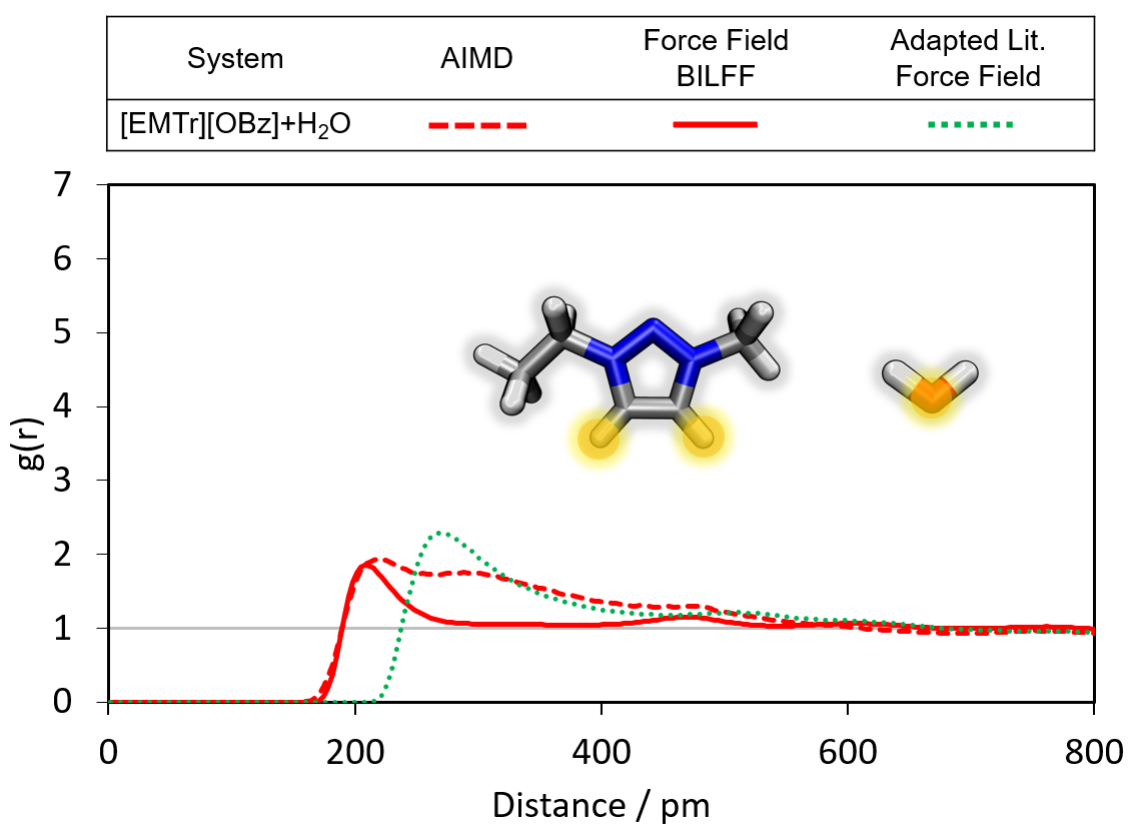
Comparison of the RDFs of the hydrogen bond between the marked oxygen atom of water and the ring protons of [EMTr]${}^{+}$ calculated from a reference AIMD and force field MD simulations of aqueous [EMTr][OBz] using adapted literature force field parameters [34,35,36,37,38,39] and BILFF. The RDFs are averaged over both ring protons.

**Figure 6:**
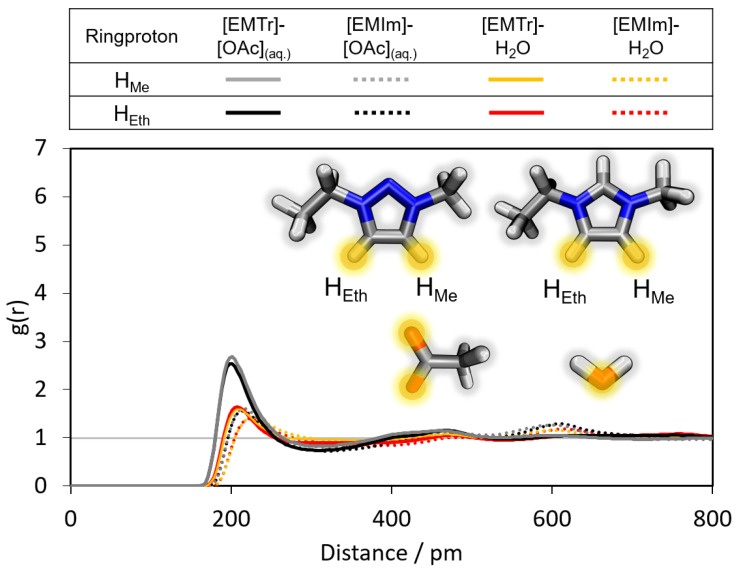
Comparison of the RDFs of the hydrogen bond between the marked oxygen atoms of [OAc]${}^{-}$/water and the ring protons of [EMIm]${}^{+}$/[EMTr]${}^{+}$ calculated from force field MD simulations of aqueous [EMTr][OAc] and [EMIm][OAc] using BILFF.

In the following, the radial distribution functions (RDFs) in the different ILs are compared with the results of the corresponding AIMD simulations. Furthermore, conclusions are drawn about the differences in the formation of hydrogen bonds between the cations [EMTr]${}^{+}$ and [EMIm]${}^{+}$ and their respective bonding partners, as well as between the anions [OBz]${}^{-}$ and [OAc]${}^{-}$ and their respective hydrogen bond donors. First, the hydrogen bonds of the cation [EMTr]${}^{+}$ are analyzed (see Figure 4, Figure 5 and Figure 6). The ring protons of [EMTr]${}^{+}$ form a strong hydrogen bond to the anion [OAc]${}^{-}$ with a particle distance of 195 pm (cf. Figure 4) and a g(r)=5.4, as can be seen from the first maximum of the RDF. The agreement of the RDF with the AIMD result is excellent. To achieve this, the partial charge of the ring protons was increased by about 27%, from 0.150 to 0.191, compared to the force field parameters for [EMIm]${}^{+}$[27]. The value of σ was decreased from 1.62 Å to 1.48 Å by about 12%, reducing the atom repulsion.

According to the height of the RDFs (Figure 5 and Figure 6), the hydrogen bond between [EMTr]${}^{+}$ and the oxygen of water is comparatively weaker than the hydrogen bond between [EMTr]${}^{+}$ and [OAc]${}^{-}$.

A comparison of the hydrogen bond of [EMTr]${}^{+}$ and H${}_{2}$O in aqueous [EMTr][OBz] between an AIMD and force field MD simulation with BILFF is shown in Figure 5. It should be noted that the force field parameters were specifically adjusted for [EMTr]${}^{+}$ in [EMTr][OAc] and [OBz]${}^{-}$ in [EMIm][OBz], but not for the combination of both ions as [EMTr][OBz]. However, the results of the RDF calculated from AIMD simulations can still be well reproduced for the [EMTr]${}^{+}$⋯H${}_{2}$O hydrogen bond, indicating the good transferability of the force field parameters for both molecules.

When comparing our results with the adapted literature force fields for [EMTr]${}^{+}$[37,38,39] and [OBz]${}^{-}$ [34,35,36] large deviations with respect to the results of the AIMD become visible, which underlines the importance of an optimization of the force field parameters for a correct reproduction of the hydrogen bonds.

Additionally, it is interesting to compare the strength of the cation–anion/cation–water hydrogen bonds of the two different aqueous ILs [EMTr][OAc] and [EMIm][OAc] shown in Figure 6. For [EMTr]${}^{+}$, the first maximum of g(r) is observed with a higher intensity at slightly smaller particle distances for both types of hydrogen bonds (cation–anion and cation–water). The ring protons of the cation [EMTr]${}^{+}$ thus form stronger hydrogen bonds to both [OAc]${}^{-}$ and water than the cation [EMIm]${}^{+}$ even though the 1,2,3-triazolium cation has a considerably weaker acidity than imidazolium ($pK_{A}$([EMTr][OAc]) = 24, $pK_{A}$([EMIm][OAc]) = 20–23) [15]. The trend persists upon an exchange of the anion ([OAc]${}^{-}$/[OBz]${}^{-}$) (cf. Figures S1 and S2 in the Supplementary Materials). One reason is the 27% higher partial charge of the ring protons of the triazolium cation due to the additional nitrogen atom in the ring in comparison to imidazolium. The reduced number of ring protons in [EMTr]${}^{+}$ also affects the competition for hydrogen bond donors, increasing the intensity of each individual interaction.

Furthermore, it was investigated whether the two aromatic ring protons in both cations differ in terms of the intensity of hydrogen bonding. The corresponding RDFs are shown in Figure 6 (black/grey curve and red/orange curve). An almost identical behavior of the curves can be observed, which occurs due to the equivalence of the ring protons in each of the two molecules.

**Figure 7:**
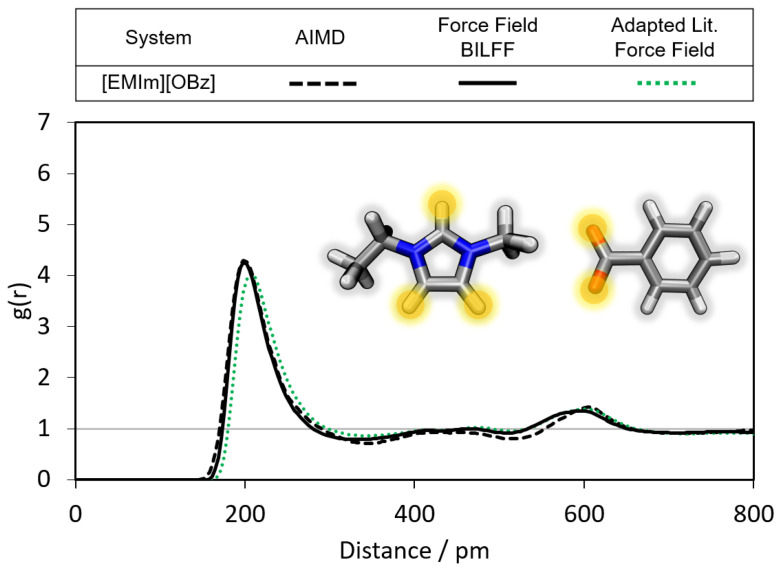
Comparison of the RDFs of the hydrogen bond between the marked oxygen atoms of [OBz]${}^{-}$ and the ring protons of [EMIm]${}^{+}$ calculated from a reference AIMD and force field MD simulations using adapted literature force field parameters [34,35,36] and BILFF. The RDFs are averaged over all three ring protons.

**Figure 8:**
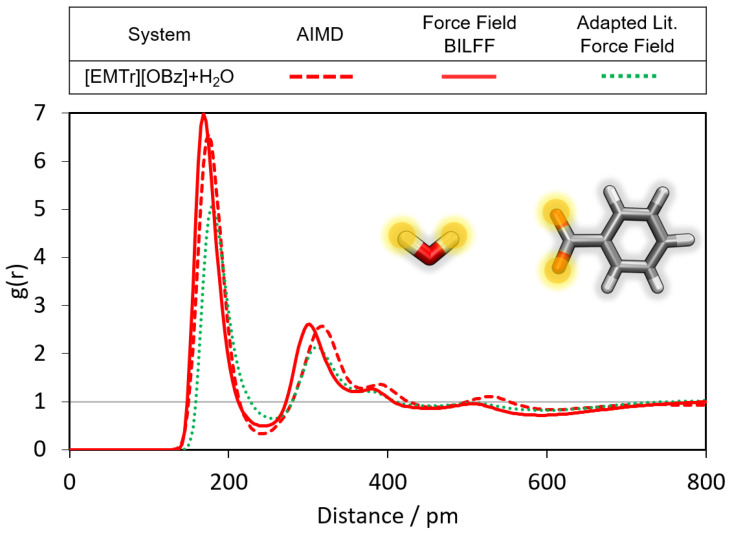
Comparison of the RDFs of the hydrogen bond between the marked oxygen atoms of [OBz]${}^{-}$ and the protons of water calculated from a reference AIMD and force field MD simulations using adapted literature force field parameters [34,35,36] and BILFF.

**Figure 9:**
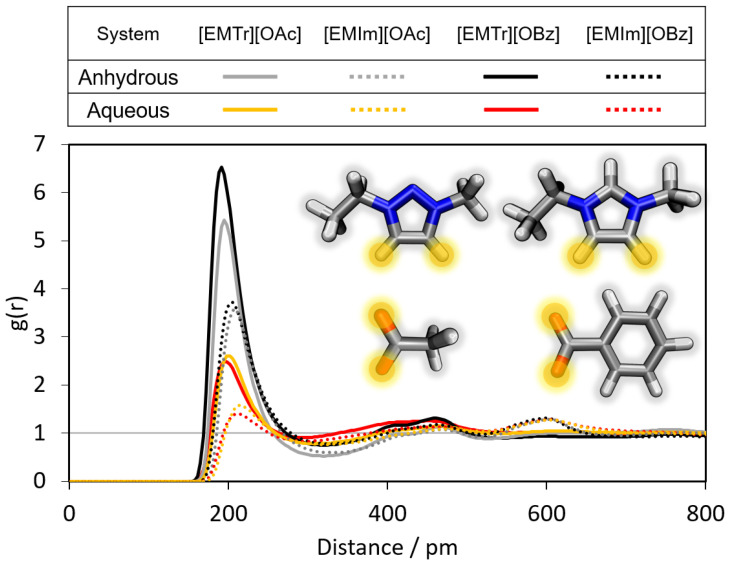
Comparison of the RDFs of the hydrogen bond between the marked oxygen atoms of [OBz]${}^{-}$ as well as [OAc]${}^{-}$ and the ring protons of [EMIm]${}^{+}$ and [EMTr]${}^{+}$ calculated from force field MD simulations using BILFF. The RDFs are averaged over the marked ring protons.

In the following, the hydrogen bonds of the second considered ion [OBz]${}^{-}$ are analyzed (cf. Figure 7, Figure 8 and Figure 9). With a g(r) of 3.7 and a particle distance of 205 pm in the first maximum of the RDF, the anion forms strong hydrogen bonds to [EMIm]${}^{+}$ (cf. Figure 7). By increasing the partial charge of the oxygen atom of [OBz]${}^{-}$ by about 5% from −0.524 to −0.550, and reducing the atom repulsion by modifying σ by about 5% from 2.96 Å to 2.80 Å compared to the adapted force field parameter of OPLS–AA [34,35,36] (cf. Table S1), an excellent agreement with the AIMD simulation was obtained. In contrast, the adapted literature force field [34,35,36] shows slight deviations in the position and height of the first maximum.

However, benzoate forms an even stronger hydrogen bond to the protons of water than to [EMIm]${}^{+}$(cf. Figure 7 and Figure 8). Besides the obvious difference in the O–H vs. C–H dipole moment of water and [EMIm]${}^{+}$, another reason can be steric effects. The oxygen atoms of benzoate are more easily accessible by the small water molecules than by the large imidazolium cation.

In comparison, the adapted literature force field gives rise to a significantly weaker peak intensity of the corresponding RDF (cf. Figure 7 and Figure 8, green curve).

In addition, a comparison of the cation–anion hydrogen bonding intensities of all four ion pair combinations is analyzed in Figure 9. The first maximum in the RDF of [OBz]${}^{-}$⋯[EMIm]${}^{+}$ is slightly increased compared to [OAc]${}^{-}$⋯[EMIm]${}^{+}$ in the anhydrous system (black and grey curves). This indicates that [OBz]${}^{-}$ forms slightly stronger hydrogen bonds to the cation than [OAc]${}^{-}$, as already observed before [40]. We can therefore confirm the previous reports in the literature. This effect is even more pronounced in the triazolium-containing ILs with a difference in the first maximum of a g(r) of 1.1. The same trend is observed when comparing the corresponding RDFs of the AIMD simulations (cf. Figure S3 in the Supplementary Materials).

In the presence of water, the respective cation⋯anion hydrogen bonds of both anion species are weakened (cf. Figure 9, red and orange curves). This is caused by the additional formation of an anion⋯water hydrogen bond, which is much stronger than the cation–anion hydrogen bond (cf. Figure S4 in the Supplementary Materials).

Due to the stronger hydrogen bond, the hydration shell around [OBz]${}^{-}$ is more pronounced than that of [OAc]${}^{-}$. As a result, there is a greater attenuation of the cation⋯[OBz]${}^{-}$ interaction relative to the cation⋯[OAc]${}^{-}$ hydrogen bond in the presence of water.

The hydration shell around the ions, as well as the coordination numbers, are discussed in Section 2.2.

In summary, the following trends are thus obtained from the comparison of the RDFs of the different ILs:

- Triazolium forms stronger hydrogen bonds with both the anion and water than imidazolium.
- Benzoate forms stronger hydrogen bonds with the cation than acetate in the anhydrous system.
- Benzoate forms stronger hydrogen bonds with water than acetate, resulting in a greater attenuation of the cation–anion interaction in the aqueous system.

#### 2.1.2. Combined Distance–Angle Distribution Functions

While radial distribution functions provide an important initial assessment of the quality of the simulated hydrogen bonding structure, the directional aspect should not be neglected.

**Figure 10:**
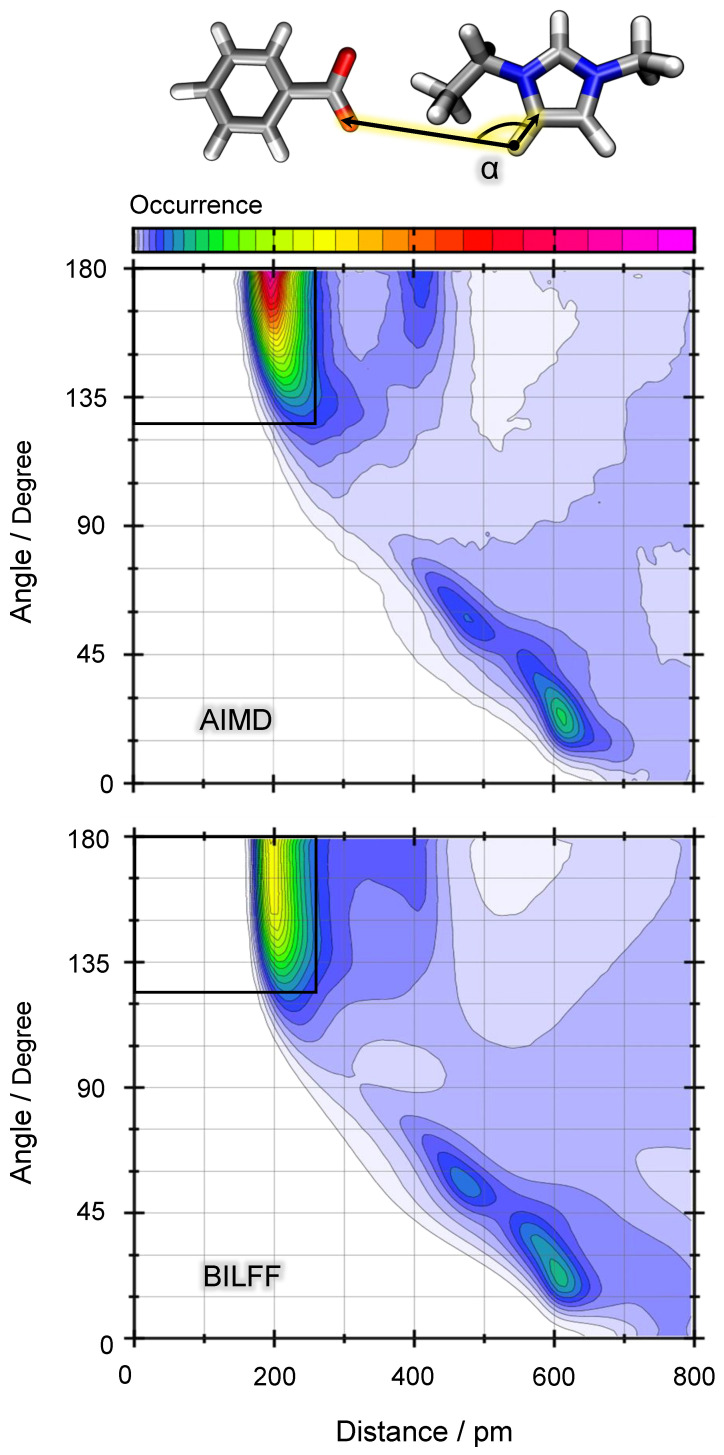
Distance–angle distribution function between an example ring proton of [EMIm]${}^{+}$ and the oxygen atoms of [OBz]${}^{-}$ in pure [EMIm][OBz] as a result of a reference AIMD simulation (**top**) and a force field MD simulation with BILFF (**bottom**). Color code in arbitrary units. The black rectangle demonstrates the geometric criterion for the existence of a hydrogen bond, as used in the lifetime calculation.

Figure 10 shows the combined distance–angle distribution functions (CDFs) of the distance H${}_{\mathrm{EMIm}}\cdots$ O${}_{\mathrm{OBz}}$ and the corresponding angle *∡*(CH${}_{\mathrm{EMIm}}$, H${}_{\mathrm{EMIm}}$, O${}_{\mathrm{OBz}}$) as illustrated in the sketch. The overall pattern of the CDFs of the AIMD simulation (top) and the FFMD simulation with BILFF (bottom) agree very well. The main hydrogen bonding peak at a particle distance of about 200 pm shows a broader angular distribution in the BILFF simulation, which may be the result of better statistical sampling compared to the AIMD simulation.

The second peak at about 400 pm originates from both the “second” oxygen of [OBz]${}^{-}$ and the “second” hydrogen of the [EMIm]${}^{+}$ molecule. Again, the AIMD simulation yields a slightly stronger localization than the force field.

The third peak, observed at approximately 600 pm, occurs at an angle of around 15${}^{\circ }$ and corresponds to the hydrogen bonding involving the isolated CH group, leading to an increased presence of benzoate ions on the “upper” side of the molecule.

Considering the CDFs of the 1,2,3-triazolium cation and the anions acetate and benzoate, a similar good agreement of the CDFs of the AIMD and FFMD simulations can be obtained (cf. Figures S8 and S9 in the Supplementary Materials). Additional CDFs regarding the cation⋯anion, cation⋯cation, and anion⋯water interactions in [EMIm][OAc] can be found in the previously published article [27].

However, for a correct reproduction of the microstructure of the AIMD simulation, not only the hydrogen bonds are important, but also the description of the π–π interactions between the aromatic rings of [EMTr]${}^{+}$ and [OBz]${}^{-}$.

**Figure 11:**
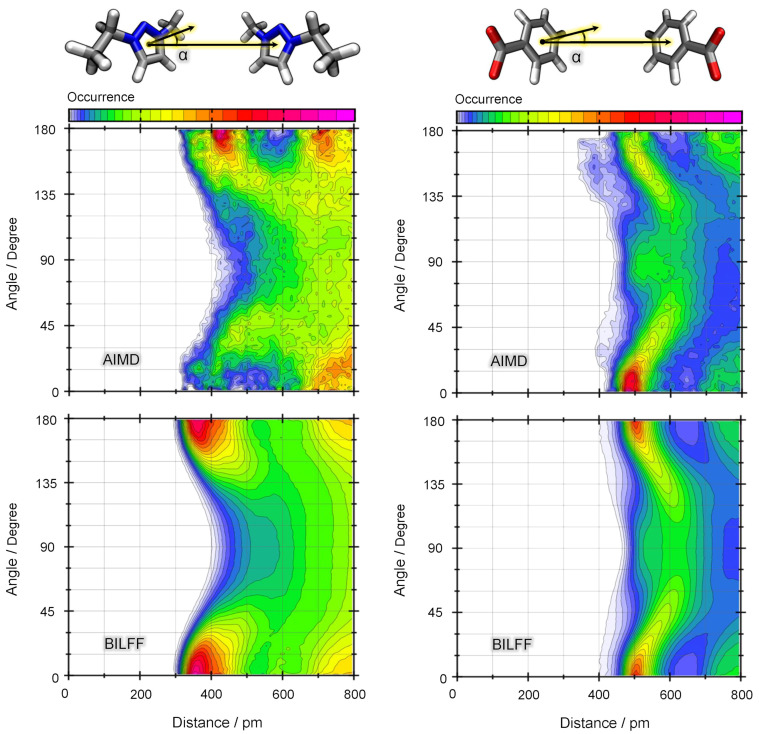
Distance–angle distribution function between two [EMTr]${}^{+}$ (**left**) and two [OBz]${}^{-}$ (**right**) ring centers in aqueous [EMTr][OBz] as a result of a reference AIMD simulation (**top**) and a force field MD simulation with BILFF (**bottom**). Color code in arbitrary units.

Considering the homomolecular distance–angle distribution functions of the aromatic cations, [EMTr]${}^{+}$ and [EMIm]${}^{+}$ (cf. Figure 11, left panel and Ref. [27]), symmetric peaks are observed at 400 pm, 0${}^{\circ }$ and 400 pm, 180${}^{\circ }$, but no residence probability is found around 400 pm, 90${}^{\circ }$. This pattern indicates the occurrence of π–π stacking. The absence of a significant probability for the cations being at a 90${}^{\circ }$ angle to each other implies that a T-shaped arrangement between the cations is unlikely.

In contrast to this, the corresponding CDF of [OBz]${}^{-}$⋯[OBz]${}^{-}$ (cf. Figure 11; right panel) shows two maxima at 500 pm for angles of 0${}^{\circ }$ and 180${}^{\circ }$, along with a broad distribution in the center region (500 pm, 90${}^{\circ }$). The unusually large distance between the molecules combined with the higher probability of $\alpha =90^{\circ }$ shows that the coplanar [OBz]${}^{-}$⋯[OBz]${}^{-}$ motif is no longer the dominant structure (as opposed to either cations). Instead, they are twisted relative to each other, adopting a T-shaped arrangement rather than a stacked configuration.

In both the anion⋯anion and cation⋯cation CDFs, the results obtained from FFMD simulations exhibit a good agreement with AIMD simulations. It is important to note here that the force field parameters were developed for [EMTr]${}^{+}$ in [EMTr][OAc] and [OBz]${}^{-}$ in [EMIm][OBz] but not for the combination [EMTr][OBz]. The fact that the microstructure of the AIMD simulation can nevertheless be reproduced well even in the case of the cross combination [EMTr][OBz] in water shows a good transferability of the force field parameters. This aspect will be discussed in more detail in Section 2.3.3.

#### 2.1.3. Spatial Distribution Functions

**Figure 12:**
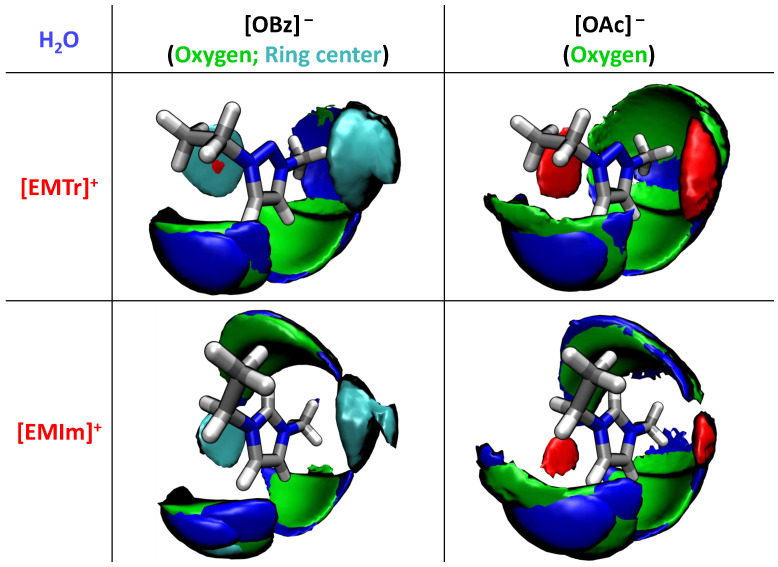
Spatial distribution function of the arrangement of molecules around the two different cations with the protons and oxygen atoms of water (blue, 26 nm${}^{-3}$) and the oxygen atom of the anion (green, 7 nm${}^{-3}$) as well as the ring center of the cation (red, 7 nm${}^{-3}$) and anion (cyan, 10 nm${}^{-3}$) in all four systems calculated with BILFF.

Providing valuable insights into the overall structural arrangement, spatial distribution functions (SDF) provide a comprehensive picture of the relative orientations of molecules within the complex system involving the different cations, anions, and water. Figure 12 shows the results of the force field MD simulation using BILFF for all four systems. The oxygen atoms of water and the anion arrange themselves competitively at the respective ring protons of the cation. Comparing the imidazolium and triazolium patterns, the isolated aromatic ring proton of [EMIm]${}^{+}$ acts as a strong attractor for the oxygen atoms, resulting in deflation around the aliphatic side chains, while the substituted nitrogen is avoided, resulting in a much more pronounced oxygen density around the methyl/ethyl side chains.

The spatial distribution function also shows distinct π–π stacking motifs above/below the aromatic cations. Interestingly, the stacking partner is determined by the anion type; for [OAc]${}^{-}$ a homomolecular cation–cation stacking (red areas in Figure 12) is observed, while for [OBz]${}^{-}$ the corresponding regions are preferentially occupied by the benzoate anions (cyan areas). The ring stacking of the benzoate is in agreement with results in the literature [16]. The results from the force field MD simulation of aqueous [EMTr][OBz] also agree well with the results obtained in the reference AIMD simulation (cf. Figure S10 in the Supplementary Materials).

### 2.2. Competing Hydrogen Bonds

**Figure 13:**
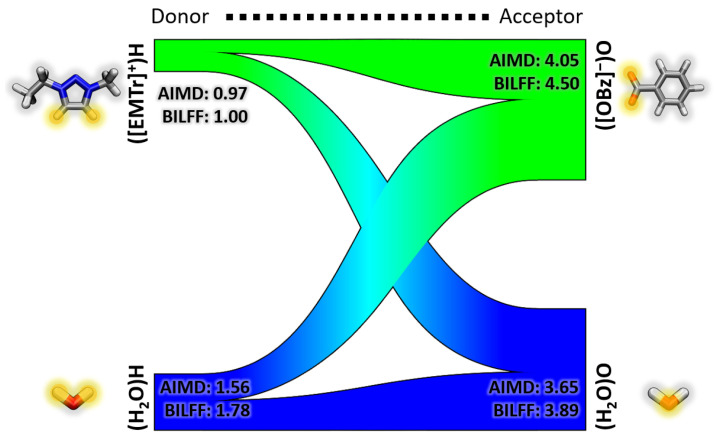
Visualization of the competing hydrogen bonds in aqueous [EMTr][OBz] as Sankey diagram calculated from a force field MD simulation with BILFF. The inserted numbers represent coordination numbers and are shown comparatively to the AIMD simulation.

In the ternary mixtures of ionic liquids in water, all direct interactions between the ions are brought into competition with hydrogen bonding interactions with water. The topology of the interaction pattern is analyzed in the form of a Sankey diagram (cf. Figure 13). The coordination numbers were calculated from the integral under the curve of the corresponding RDFs to the first minimum. These numbers indicate the number of hydrogen bonds per atom, which is proportional to the thickness of the bars in the Sankey diagram.

The triazolium cation exhibits a coordination number of 1.0 in aqueous [EMTr][OBz] (cf. Figure 13), and 1.21 in aqueous [EMTr][OAc] (cf. Figure S11 in the Supplementary Materials). The imidazolium cation has similar coordination numbers of 0.95 in aqueous [EMIm][OBz] and 1.15 in aqueous [EMTr][OAc] (cf. Figures S12 and S13 in the Supplementary Materials).

Considering the anions, benzoate is surrounded by about 4.5 other molecules of the cation or water in the corresponding ILs (cf. Figure 12 and Figure S12 in the Supplementary Materials). The smaller acetate, on the other hand, has a slightly higher coordination number of about 5 (cf. Figure 12 and Figure S12 in the Supplementary Materials).

Compared to the results for [EMTr][OBz] from the AIMD simulation, the coordination numbers of BILFF can be well reproduced, especially for the cation. It should be noted, that the force field parameters were not explicitly optimized for this combination, but for [EMTr][OAc] and [EMIm][OBz].

### 2.3. Temperature Dependence

#### 2.3.1. Radial Distribution Functions at Higher Temperatures

**Figure 14:**
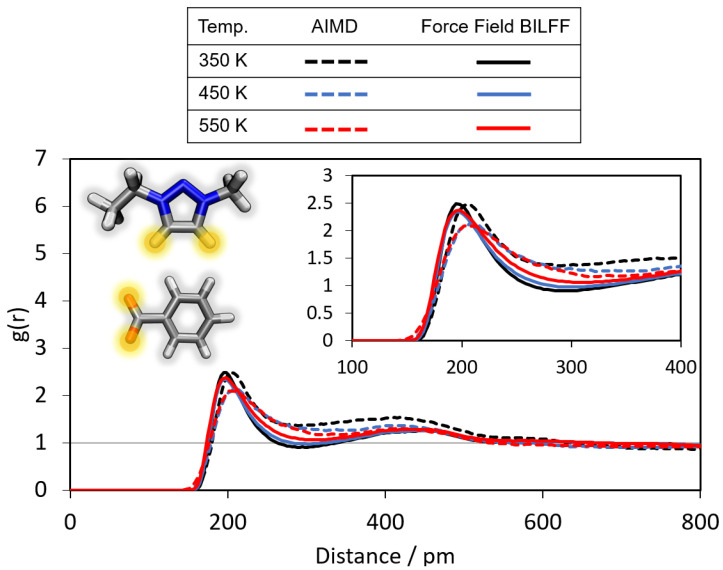
Radial distribution functions (RDFs) of the hydrogen bonds between the marked oxygen atoms of [OBz]${}^{-}$ and the ring protons of [EMTr]${}^{+}$ in aqueous [EMTr][OBz] at different temperatures, simulated by AIMD simulation and force field MD simulation using BILFF.

To verify the transferability of BILFF to [EMTr][OBz] force field MD simulations were performed and compared with reference AIMD simulations. Also, the application of the force field at elevated temperatures has been investigated and is discussed in this section. The radial distribution functions (RDFs) of the hydrogen bonds between the ring protons of the cation and the oxygen atoms of the anion are shown in Figure 14.

At 350 K, the first maximum of the RDF occurs at a particle distance of 205 pm and a g(r)=2.5. As expected, with increasing temperature, the maximum decreases slightly to a g(r)=2.1 at a temperature of 550 K. As the temperature rises, the mobility of the molecules increases, so that the hydrogen bonds become more short-lived, resulting in a smaller g(r).

Similar to the AIMD simulation, the height of the maxima in the RDFs of the force field MD simulation does not change drastically with increasing temperature. Although the force field parameters were not directly optimized for aqueous [EMTr][OBz], the agreement with the reference AIMD simulation is very good, even at high temperatures, underlining the high accuracy of the optimized force field parameters of BILFF.

#### 2.3.2. Hydrogen Bond Lifetime

Up to this point, a number of structural quantities have been computed, mainly expressed as distribution functions. Exemplary dynamical properties have also been calculated, in particular the hydrogen bond lifetime.

In Table 6, the hydrogen bond lifetime calculated from the AIMD and FFMD simulation using BILFF are compared. Furthermore, a comparative analysis of the lifetimes of the different hydrogen bonds in the investigated ILs [EMTr][OAc], [EMIm][OBz] and [EMTr][OBz] is shown. In addition, a comparison with the IL [EMIm][OAc] is made. The lifetime of the hydrogen bonds is studied in the presence and absence of water.

The calculation of the lifetime is differentiated in the intermittent and continuous calculation whereby, in the intermittent calculation, the breaking and reforming of the hydrogen bond is allowed. The continuous lifetime, on the other hand, represents the duration until the very first breakage of the hydrogen bond. To calculate the lifetime, geometric criteria were defined on the basis of the first maximum in the underlying distance–angle distribution functions. These criteria are listed in Table S5 in the Supplementary Materials. In Figure 10, the definition of the geometric criterion was exemplified as a black rectangle. The lifetime values presented in Table 6 are, respectively, averaged over all ring protons of the imidazolium (three ring protons) and 1,2,3-triazolium cation (two ring protons). The lifetime of the hydrogen bonds of the single protons H${}_{\mathrm{Me}}$ and H${}_{\mathrm{Eth}}$ can be found in Table S6 in the Supplementary Materials. (The lifetime of the hydrogen bonds [EMIm]${}^{+}$⋯[OAc]${}^{-}$ as well as [EMIm]${}^{+}$⋯water, with respect to all three individual ring protons can be found in the previous article on BILFF [27]).

The following conclusions can be drawn:

The longest hydrogen bond lifetime is observed in the anhydrous system, primarily due to strong Coulomb interactions, with [EMTr]${}^{+}$ forming more durable hydrogen bonds with the anion than [EMIm]${}^{+}$. However, this trend is reversed in the aqueous ILs.

The introduction of water results in the reduction of the hydrogen bond lifetime between the cations and anions in all ILs, due to a competition for oxygen atoms surrounding the cation, as well as a lower viscosity. This competition between water and the anion can also be observed in the radial and spatial distribution functions (see Section 2.1.1 and Section 2.1.3) as well as in the Sankey diagrams (see Section 2.2). At the same time, the anion [OBz]${}^{-}$ forms more long-lasting hydrogen bonds to water than [OAc]${}^{-}$. This is also reflected in the frequency of the occurrence of the hydrogen bond of the ILs measured by the height of the first maximum of the RDF.

However, the lifetime of the hydrogen bonds can be affected not only by water, but also by an increased temperature. Due to the resulting higher mobility of the molecules, the lifetime of the hydrogen bonds decreases, as seen in aqueous [EMTr][OBz].

In all four ILs, the first breakup of the hydrogen bonds take place after only a few picoseconds (about 1–7 ps). Therefore, the two cation ring protons H${}_{\mathrm{Me}}$ and H${}_{\mathrm{Eth}}$ show similar values of lifetime (see Table S6 in the Supplementary Materials). In BILFF, they are regarded as equivalent.

It should be noted that dynamical properties, such as lifetimes, depend exponentially on the potential energy surface and are therefore subject to larger variations (with respect to the experiment, but also with respect to variations of the theoretical methods). From this perspective, the agreement between the force field MD simulation with BILFF and the AIMD simulation is still very good. It is also noteworthy that some of the lifetimes are in excess of the total simulation time of the AIMD simulation.

#### 2.3.3. Validation of the Density and Diffusion

To test the reproducibility of experimental quantities, one approach is to compare the calculated system densities of the different ILs with corresponding experimental data (see Table 7). As expected, a decrease in density is observed in the presence of water. Pure [EMTr][OBz] has the highest density and (aqueous) [EMIm][OAc] the lowest. This difference can be attributed to the larger molecular volume occupied by [EMTr]${}^{+}$ and [OBz]${}^{-}$. The agreement of the density of pure [EMTr][OAc] as well as of the combined IL [EMTr][OBz] for which the force field parameters were not specifically optimized, is outstanding. The deviation is only 0.9% from the experimental measurement. The experimental density of [EMIm][OBz] is reproduced without any deviation at all.

The mobility of the molecules also plays a crucial role in accurately describe the behavior of the ILs. Below, the self-diffusion coefficients in all investigated ionic liquids calculated from the force field MD simulations are compared with the reference AIMD simulation (see Table 8). The following observations can be made:

- According to the FFMD results, the bulky cation generally diffuse faster than the anion [42]. An exception is [EMIm][OAc] in water, where the anion diffuses faster. This phenomenon is already known and is in agreement with experimental data for [EMIm][OAc] [43].
- The diffusion coefficient is higher at elevated temperatures.
- In the presence of water, the diffusion coefficient of all ionic liquids increases significantly.
- The diffusion coefficient is influenced by both the cations and anions in the IL. When comparing the diffusion of the cations in the different ILs containing either [EMTr]${}^{+}$ or [EMIm]${}^{+}$, it is observed that the triazolium cations exhibit a slower diffusion compared to imidazolium. Furthermore, when comparing the diffusion rates of the anions, it is found that benzoate diffuses slower than acetate in both pure and aqueous systems. This is due to the different occupied volumes and thus the bulkiness of the molecules as well as their more pronounced hydrogen bond formation.
- The lowest diffusion rate, and, at the same time, the highest density, can be found in pure [EMTr][OBz].

The results of the force field MD simulations reflect the trends of the reference AIMD simulations for the imidazolium-containing ILs. In the case of the triazolium-containing ILs, the AIMD simulation shows a slightly faster diffusion of the anion compared to the cation at 350 K and 450 K, and thus an opposite trend to the FFMD. Despite occasional numerical fluctuations in the specific diffusion values, the overall agreement of this dynamic quantity with the first-principles reference simulation is very good. The temperature dependence is reproduced over more than one order of magnitude. It should be noted that [EMTr][OBz] has been synthesized recently, so no experimental data for the diffusion coefficient are available yet. For [EMIm][OAc] the diffusion coefficient agrees very well with experimental data ($D_{[\mathrm{EMIm}],\;\exp.}=14.1\;\cdot \;\;10^{-11}$ m${}^{2}$ s${}^{-1};D_{[\mathrm{OAc}],\;\exp.}=12.8\;\cdot \;\;10^{-11}\;$m${}^{2}$ s${}^{-1}$) [44].

## 3. Computational Details

In this section, the computational details of the underlying reference AIMD simulations and force field MD simulations are presented. As a starting configuration for the AIMD simulations, the last time step of a force field MD simulation using the bonded and Lennard-Jones parameters of OPLS–AA [34,35,36] and the CL&P force field [37,38,39] and RESP partial charges (see Ref. [15]) as well as TIP4P–EW [45] (with constrained bonds and angles using the SHAKE algorithm [46,47]) for water was used. The physical simulation time was 5 ns for [EMIm][OBz], 0.5 ns for [EMTr][OAc] and 20 ns for aqueous [EMTr][OBz]. The final AIMD simulations were performed in analogy to the previous articles on BILFF [27,28] with CP2k [48,49,50] using the quickstep method [51] and orbital transformation (OT) [52]. Under application of the density functional theory [53,54] using the BLYP functional [55,56], dispersion correction D3(BJ) of Grimme [57,58] and the revised damping parameters of Smith et al. [59], the electron density was calculated. The DZVP-MOLOPT-SR-GTH [60] and GTH pseudopotentials [61,62] with a plane-wave energy cutoff of 350 Ry were used as basis sets. To equilibrate the AIMD simulations, the first 30 ps for [EMTr][OAc] and the first 50 ps for the systems with [OBz]${}^{-}$ were discarded, respectively. The physical simulation time, size of the simulation box, and particle numbers can be found in Table 9.

The force field MD simulations for optimizing the force field parameter were also performed analogously to the previous articles on BILFF [27,28]. After constructing the simulation box using Packmol [63], an equilibration was performed at 500 K in the *NVT* ensemble using a Berendsen thermostat [64] (coupling constant of 1.0 fs) in a 25 ps simulation followed by a 100 ps simulation at a temperature of 350 K in the *NpT* ensemble using a Nosé–Hoover thermostat [65,66,67] (coupling constant of 100 fs) and a Nosé–Hoover barostat (coupling constant of 2000 fs). Using the Langevin thermostat [68,69], the acoustic shock waves were damped after the equilibration runs and adjustment of the final box volume. The production run was finally computed in the *NVT* ensemble using the Nosé–Hoover thermostat [65,66,67] (coupling constant of 100 fs) and a time integrator of 0.5 fs. The PPPM long-range solver of LAMMPS [70] was applied with a Lennard-Jones cutoff radius of 800 pm to calculate the electrostatic interactions.

As a starting point for the optimization of the force field parameters BILFF [27,28] for [EMTr]${}^{+}$ was used. The reference bond length and angle of atom type NR were determined using a geometry optimization. For [OBz]${}^{-}$, the Lennard-Jones parameters and the force constants were adapted from OPLS–AA [34,35,36]. The partial charges were calculated from quantum chemical calculations via the restrained electrostatic potential (RESP) methodology. The equilibrium bond length was calculated using a geometry optimization. The starting force field parameters are listed in the Supplementary Materials.

The ionic charge was set to ±0.82, as in the force field development of [EMIm][OAc] [27]. Reducing the total ion charge is discussed as an alternative to the usage of computationally more expensive polarizable force fields in the literature and is widely applied [71,72,73,74,75].

To validate the force field, additional force field MD simulations of pure and aqueous [EMTr][OBz] at 350 K as well as force field and reference AIMD simulations of aqueous [EMTr][OBz] at elevated temperatures of 450 K and 550 K were calculated. It should be noted that, for this IL, the force field parameters have not been optimized and the transferability of the parameters for [EMTr]${}^{+}$ in [EMTr][OAc] and [OBz]${}^{-}$ in [EMIm][OBz] to this IL will be tested. The same simulation parameters as described above were used. The first 30 ps of the AIMD simulations were again discarded for equilibration. The resulting physical simulation time is 88 ps in each case. An analysis of the temperature dependence of the simulations can be found in Section 2.3.

To analyze the trajectories, the program package TRAVIS [76,77] was applied. Xmgrace [78], Wolfram Mathematica [79], and VMD [80] with the Tachyon renderer [81] were used to create the figures.

## 4. Conclusions

In this article, we present the extension of our all-atom force field BILFF (Bio-Polymers in Ionic Liquids Force Field) to the ionic liquids [EMTr][OAc], [EMIm][OBz] and [EMTr][OBz] in absence and presence of water. BILFF was previously tuned for an optimal balance of the competing interactions in the ternary mixture of [EMIm][OAc] and water in view of its application to the challenge of modeling the solvation structure of cellulose (as solute) by [EMIm][OAc]/water [27,28]. The focus of this work is the generalization of the [EMIm][OAc] force field to additional ions ([EMTr]${}^{+}$, [OBz]${}^{-}$) including all four combinations.

This article thus presents the first force field specifically optimized for [EMTr][OAc] and [EMIm][OBz]. The objective was to ensure an accurate reproduction of the quantum chemical microstructure as well as dynamic properties such as hydrogen bond lifetimes and diffusion in these ILs. To achieve this, an iterative adjustment of the force field parameters to the results of reference AIMD simulations and experimental system densities was performed. In addition, to verify the transferability of the optimized force field parameters for [EMIm][OBz] and [EMTr][OAc], the results of (aqueous) [EMTr][OBz] were evaluated.

By comparing the simulation results of the four ILs [EMTr][OAc], [EMIm][OAc], [EMTr][OAc] and [EMTr][OBz], the hydrogen bonding strength of benzoate was found to be slightly stronger than that of acetate, in spite of the smaller partial charges on the oxygen atoms (−0.55e vs. −0.66e). This trend is inverted by water. This observation is surprising at first glance, and indeed we cannot provide a “single” answer to this phenomenon. One contributing factor to this enhancement of hydrogen bonding strength is the reduced rotational/translational freedom due to the T-formation of the anions, which leads to a more stable spatial arrangement and a longer lifetime of the hydrogen bonds. Regarding the cations, the hydrogen bond strength is stronger for triazolium compared to imidazolium. With the CH/N substitution as the only molecular difference, the enhancement is most likely due to the higher partial charges of the CH ring protons (+0.19e vs. +0.15e). This different ionic hydrogen bonding strengths play an ambiguous role, in view of the solubility of cellulose in the different ILs; while a good hydrogen bonding ability (mainly of the anions) is essential to break the cellulose–cellulose bonds, this effect is counterbalanced by an equally increased hydrogen bond strength between the ion pairs. These two effects are in competition with each other, and again, no “single argument” is able to resolve this issue unambiguously.

Experimentally, the solubility of cellulose increases in the following order: [EMIm][OBz] < [EMTr][OBz] < [EMTr][OAc] < [EMIm][OAc] [15,16]. The results of the simulations show that the best solvent [EMIm][OAc] has the weakest cation–anion interaction in its pure form, whereas the IL with the strongest ion pair interactions, [EMTr][OBz], is the second poorest cellulose solvent. However, the even lower solubility in [EMIm][OBz] cannot be explained by this scheme, as its cation–anion interaction is somewhat weaker than that of [EMTr][OBz].

Clearly, further factors must be considered to establish a complete picture of cellulose solubility in such complex solvents. One important aspect is the mere spatial size of the anion, as it strongly influences its diffusivity and thus its ability to intercalate into the cellulose crystal/fibrils and initiate the disruption of the internal cellulose hydrogen bonding network. Another point to consider is the influence of the van der Waals effects, which also alter the balance of the competing interactions between the anion/cation/water/cellulose molecules.

## Figures and Tables

**Table 1:** Nomenclature of the atom types and atom classes of [EMTr]${}^{+}$ and [OBz]${}^{-}$ in BILFF. The atom types are applied for the non-bonded interactions (see Table 2), while the atom classes are used for the bonded interactions (see Table 3, Table 4 and Table 5).

Atom Type	Atom Class
[EMTr]${}^{+}$	
C1	CT
CE	CT
CM	CT
CW	CW
HCW	HA
H1	HC
HE	HC
HM	HC
NR	NR
NA	NA
[OBz]${}^{-}$	
C1	CA
C2	CA
C3	CA
C4	CA
CO	CO
H2	HA
H3	HA
H4	HA
O2	O2

**Table 2:** Optimized atomic partial charges *q* and Lennard-Jones parameters σ and ϵ of [EMTr]${}^{+}$ and [OBz]${}^{-}$ in BILFF.

Atom Type	*q*	σ	ϵ
	/ e	/ Å	/ kJ mol${}^{-1}$
[EMTr]${}^{+}$			
C1	−0.187	3.34	0.2760
CE	−0.054	3.34	0.2760
CW	−0.144	3.38	0.2930
HCW	0.191	1.48	0.1260
HC	0.070	2.38	0.1260
H1	0.148	2.38	0.1260
NR	−0.204	3.10	0.7110
NA	0.204	3.10	0.7110
[OBz]${}^{-}$			
C1	0.005	3.70	0.2929
C2	−0.118	3.70	0.2929
C3	−0.121	3.70	0.2929
C4	−0.299	3.70	0.2929
CO	0.398	3.90	0.4393
H2	0.070	2.42	0.1255
H3	0.157	2.42	0.1255
H4	0.200	2.42	0.1255
O2	−0.550	2.80	0.8786

**Table 3:** Optimized bond equilibrium lengths $l_{0}$ and force constants $k_{l}$ of [EMTr]${}^{+}$ and [OBz]${}^{-}$ in BILFF.

Bond	$l_{0}$	$k_{l}$
	/ Å	/ kJ mol${}^{-1}\;{Å}^{-2}$
[EMTr]${}^{+}$		
NA–NR	1.344	3199.2
CW–HA	1.088	2633.8
CW–NA	1.375	3108.7
CW–CW	1.386	3773.2
NA–CT	1.488	2046.3
HC–CT	1.099	2679.4
CT–CT	1.533	2125.5
[OBz]${}^{-}$		
CA–CA	1.387	3274.1
CA–HA	1.088	2707.4
CA–CO	1.504	1906.9
CO–O2	1.282	4273.1

**Table 4:** Optimized angle equilibrium values $\theta_{0}$ and force constants $k_{\theta }$ of [EMTr]${}^{+}$ and [OBz]${}^{-}$ in BILFF.

Angle	$\theta_{0}$	$k_{\theta }$
	/ Deg	/ kJ mol${}^{-1}$ rad${}^{-2}$
[EMTr]${}^{+}$		
CW–NA–NR	112.1	568.7
NR–NA–CT	118.6	396.5
NA–NR–NA	104.4	610.1
NA–CT–CT	110.9	361.2
NA–CW–CW	107.0	579.7
NA–CW–HA	120.8	200.9
CW–CW–HA	131.7	190.5
NA–CT–HC	107.2	375.9
CT–CT–HC	111.4	296.2
HC–CT–HC	109.2	226.5
CW–NA–CT	125.2	242.9
[OBz]${}^{-}$		
CA–CA–CA	120.0	446.0
CA–CA–HA	120.0	258.1
CA–CA–CO	120.0	397.6
CA–CO–O2	117.0	550.2
O2–CO–O2	126.0	735.9

**Table 5:** Optimized torsional coefficients $V_{n}$ of [EMTr]${}^{+}$ and [OBz]${}^{-}$ in BILFF.

Torsion Angle	$V_{1}$	$V_{2}$	$V_{3}$	$V_{4}$
	/ kJ mol ${}^{-1}$	/ kJ mol ${}^{-1}$	/ kJ mol ${}^{-1}$	/ kJ mol ${}^{-1}$
[EMTr]${}^{+}$				
CW–NA–NR–NA	0.0000	19.4600	0.0000	0.0000
CT–NA–NR–NA	0.0000	19.4600	0.0000	0.0000
NR–NA–CW–CW	0.0000	12.5500	0.0000	0.0000
NR–NA–CW–HA	0.0000	12.5500	0.0000	0.0000
NR–NA–CT–HC	0.0000	0.0000	0.0000	0.0000
NR–NA–CT–CT	0.1000	1.0000	0.1000	−0.3000
CT–NA–CW–CW	0.0000	12.5500	0.0000	0.0000
CT–NA–CW–HA	0.0000	12.5500	0.0000	0.0000
NA–CW–CW–NA	0.0000	65.0000	0.0000	0.0000
NA–CW–CW–HA	0.0000	44.9800	0.0000	0.0000
HA–CW–CW–HA	0.0000	30.0000	0.0000	0.0000
CW–NA–CT–HC	0.1000	0.2000	0.0000	0.0000
CW–NA–CT–CT	0.4000	1.0000	0.0000	0.2000
NA–CT–CT–HC	0.0000	0.0000	0.3670	0.0000
HC–CT–CT–HC	0.0000	0.0000	1.2552	0.0000
[OBz]${}^{-}$				
CA–CA–CA–CA	0.0000	30.334	0.0000	0.0000
HA–CA–CA–CA	0.0000	30.334	0.0000	0.0000
HA–CA–CA–CO	0.0000	30.334	0.0000	0.0000
HA–CA–CA–HA	0.0000	30.334	0.0000	0.0000
CA–CA–CA–CO	0.0000	30.334	0.0000	0.0000
CA–CA–CO–O2	0.0000	8.000	0.0000	0.0000

**Table 6:** Overview of the lifetime τ of the hydrogen bonds in all four systems comparing the results of the reference AIMD simulation and the force field MD simulation using BILFF at the given temperatures (C${}^{+}=$ Cation, A${}^{-}=$ Anion). (No AIMD simulations of anhydrous [EMTr][OBz] as well as [EMTr][OAc]/H${}_{2}$O and [EMIm][OBz]/H${}_{2}$O have been performed, so no data are available for these).

Temp.	Intermittent		Continuous	
	τ (AIMD)	τ (FFMD)	τ (AIMD)	τ (FFMD)
/ K	/ ps	/ ps	/ ps	/ ps
[EMTr][OAc]				
(C${}^{+}$)H⋯O(A${}^{-}$)				
350	627.1	855.3	4.0	4.1
[EMTr][OAc]/H${}_{2}$O				
(C${}^{+}$)H⋯O(A${}^{-}$)				
350	–	117.6	–	1.9
(C${}^{+}$)H⋯O(H${}_{2}$O)				
350	–	32.9	–	1.0
(H${}_{2}$O)H⋯O(A${}^{-}$)				
350	–	58.3	–	0.2
[EMIm][OAc] ${}^{a}$				
(C${}^{+}$)H⋯O(A${}^{-}$)				
350	472.0	779.7	3.0	4.5
[EMIm][OAc]/H${}_{2}$O ${}^{a}$				
(C${}^{+}$)H⋯O(A${}^{-}$)				
350	73.2	146.0	1.3	1.8
(C${}^{+}$)H⋯O(H${}_{2}$O)				
350	31.2	40.4	0.6	0.8
(H${}_{2}$O)H⋯O(A${}^{-}$)				
350	153.2	165.7	0.2	0.8
[EMIm][OBz]				
(C${}^{+}$)H⋯O(A${}^{-}$)				
350	242.2	1841.6	1.0	2.0
[EMIm][OBz]/H${}_{2}$O				
(C${}^{+}$)H⋯O(A${}^{-}$)				
350	–	207.3	–	1.2
(C${}^{+}$)H⋯O(H${}_{2}$O)				
350	–	236.0	–	1.7
(H${}_{2}$O)H⋯O(A${}^{-}$)				
350	–	299.7	–	6.6
[EMTr][OBz]				
(C${}^{+}$)H⋯O(A${}^{-}$)				
350	–	2821.1	–	6.3
[EMTr][OBz]/H${}_{2}$O				
(C${}^{+}$)H⋯O(A${}^{-}$)				
350	105.0	203.8	1.6	2.5
450	25.5	27.8	0.8	1.4
550	15.5	11.2	0.6	1.0
(C${}^{+}$)H⋯O(H${}_{2}$O)				
350	38.1	48.3	0.7	1.0
450	5.7	6.4	0.4	0.6
550	–	2.3	0.3	0.5
(H${}_{2}$O)H⋯O(A${}^{-}$)				
350	466.6	273.1	4.4	8.8
450	36.7	34.8	1.0	2.2
550	15.6	11.4	0.5	1.3

${}^{a}$ Calculated from the MD simulations of our already published article [27].

**Table 7:** Comparison of the system densities from force field MD simulations using BILFF at 350 K. The molar fraction of the ionic liquids in the aqueous systems is $\chi_{\mathrm{IL}}=0.25$.

System	Temp.	Box Size	ρ(FFMD)	ρ(Lit.)
	/ K	/ pm	/ g cm${}^{-3}$	/ g cm${}^{-3}$
[EMTr][OAc]	350	3198	1.11	1.12 ${}^{a}$
[EMTr][OAc]/H${}_{2}$O	350	3027	1.09	–
[EMIm][OAc]	350	3225	1.08 ${}^{b}$	1.07 ${}^{c}$
[EMIm][OAc]/H${}_{2}$O	350	3043	1.07 ${}^{b}$	1.07 ${}^{c}$
[EMIm][OBz]	350	3550	1.10	1.10 ${}^{d}$
[EMIm][OBz]/H${}_{2}$O	350	3286	1.09	–
[EMTr][OBz]	350	3529	1.13	1.14 ${}^{e}$
[EMTr][OBz]/H${}_{2}$O	350	3271	1.10	–
	450	3369	1.01	–
	550	3502	0.90	–

${}^{a}$ Measurements at 323.15 K from Ref. [15]. ${}^{b}$ Calculated from the MD simulations of our already published article [27]. ${}^{c}$ Extrapolated values of temperature dependent measurements from Ref. [41] (x${}_{\mathrm{IL}}$ = 0.252 in the aqueous system). ${}^{d/e}$ Measurements at 358.15 K from Ref. [16].

**Table 8:** Self-diffusion coefficients *D* from force field MD simulations using BILFF. For comparison, the diffusion coefficients of [EMIm][OAc] are also shown from our previously published article [27].

Molecule	Temp.	*D*(AIMD)	*D*(FFMD)
	/ K	/ $10^{-11}$ m${}^{2}$ s${}^{-1}$	/ $10^{-11}$ m${}^{2}$ s${}^{-1}$
[EMTr][OAc]			
[EMTr]${}^{+}$	350	4.02	5.94
[OAc]${}^{-}$		4.79	5.35
[EMTr][OAc]/H${}_{2}$O			
[EMTr]${}^{+}$	350	–	24.42
[OAc]${}^{-}$		–	22.15
H${}_{2}$O		–	54.88
[EMIm][OAc] ${}^{a}$			
[EMIm]${}^{+}$	350	7.09	9.26
[OAc]${}^{-}$		6.61	6.72
[EMIm][OAc]/H${}_{2}$O ${}^{a}$			
[EMIm]${}^{+}$	350	24.95	21.77
[OAc]${}^{-}$		28.10	22.42
H${}_{2}$O		46.88	55.97
[EMIm][OBz]			
[EMIm]${}^{+}$	350	10.33	3.72
[OBz]${}^{-}$		8.92	1.89
[EMIm][OBz]/H${}_{2}$O			
[EMIm]${}^{+}$	350	–	16.01
[OBz]${}^{-}$		–	11.42
H${}_{2}$O		–	42.83
[EMTr][OBz]			
[EMTr]${}^{+}$	350	–	1.77
[OBz]${}^{-}$		–	1.13
[EMTr][OBz]/H${}_{2}$O			
[EMTr]${}^{+}$	350	9.41	15.30
	450	96.73	123.40
	550	244.91	370.79
[OBz]${}^{-}$	350	11.75	10.54
	450	97.40	102.42
	550	186.37	310.49
H${}_{2}$O	350	25.56	48.66
	450	252.32	358.21
	550	562.49	102.76

${}^{a}$ Calculated from the MD simulations of our already published article [27].

**Table 9:** Simulation parameters and physical simulation time (sim. time) of the final equilibrated ab initio and force field MD simulations of [EMIm][OBz] and aqueous [EMTr][OBz] for the development and validation of the force field.

System	Composition	Sim. Time	Box Size	Density
		/ ps	/ pm	/ g cm${}^{-3}$
		AIMD		
[EMTr][OAc]	36 [EMTr][OAc]	30 + 223	2121	1.072
[EMIm][OBz]	36 [EMIm][OBz]	50 + 46	2319	1.114
[EMTr][OBz]	27 [EMTr][OBz]	50 + 103	2319	1.033
/H${}_{2}$O	81 Water			
		FFMD		
[EMTr][OAc]	128 [EMTr][OAc]	10,000	3198	1.113
[EMTr][OAc]	81 [EMTr][OBz]	10,000	3027	1.092
/H${}_{2}$O	243 Water			
[EMIm][OBz]	128 [EMIm][OBz]	10,000	3550	1.103
[EMIm][OBz]	81 [EMTr][OBz]	10,000	3286	1.086
/H${}_{2}$O	243 Water			
[EMTr][OBz]	128 [EMTr][OBz]	10,000	3529	1.128
[EMTr][OBz]	81 [EMTr][OBz]	10,000	3271	1.105
/H${}_{2}$O	243 Water			

## Data Availability

The data used to support the results of this study are included in the article. The corresponding author [M.B.] can be contacted for available data supporting the findings.

## Supplementary Materials

The following supporting information can be downloaded at: https://www.mdpi.com/article/10.3390/molecules28227592/s1. Tables S1–S4: Comparison of the force field parameter of [EMTr]${}^{+}$ and [OBz]${}^{-}$ in BILFF and the adapted literature force field; Figures S1–S6: Additional radial distribution functions; Figures S7 and S8: Additional distance–angle combined distribution functions; Figure S9: Spatial distribution function of aqueous [EMTr][OBz] resulted from a force field MD simulation using BILFF and the reference AIMD simulation; Figures S10–S12: Sankey diagrams of aqueous [EMTr][OAc], [EMIm][OAc] and [EMIm][OBz]; Table S5: Angle and distance criteria of the different hydrogen bonds for the calculation of the hydrogen bond lifetime; Table S6: Overview of the lifetime of the hydrogen bonds in [EMTr][OAc], [EMIm][OAc], [EMIm][OBz] and [EMTr][OBz] in absence and presence of water comparing the results of the reference AIMD simulation and the force field MD simulation using BILFF.

## Abbreviations

The following abbreviations are used in this article:

AIMD	Ab initio Molecular Dynamics
BILFF	Bio-polymers in Ionic Liquids Force Field
CDF	Combined Distribution Function
FFMD	Force Field Molecular Dynamics
IL	Ionic Liquid
MD	Molecular Dynamics
RDF	Radial Distribution Function
SDF	Spatial Distribution Function
