# Supplementary material for: BILFF: All-Atom Force Field for Modeling Triazolium- and Benzoate-Based Ionic Liquids
Source: Molecules. 2023 Nov 14;28(22):7592. doi: 10.3390/molecules28227592 (PMC10674667; doi:10.3390/molecules28227592)
Supplement: Supplementary file 1 [file molecules-28-07592-s001.zip › molecules-2637542-supplementary.pdf]

BILFF: All-Atom Force Field for Modeling Triazolium- and Benzoate-Based Ionic Liquids

Supporting Information

Eliane Roos [1], Daniel Sebastiani [1] and Martin Brehm [1,2], *

1 *Institut für Chemie—Theoretische Chemie, Martin-Luther-Universität Halle–Wittenberg, Von-Danckelmann-Platz 4, 06120 Halle (Saale), Germany; eliane.roos@gmx.de (E.R.); daniel.sebastiani@chemie.uni-halle.de (D.S.)*

2 Department Chemie, Universität Paderborn, Warburger Straße 100, 33098 Paderborn, Germany

* Correspondence: martin_brehm@gmx.de

1 Force Field Parameters

This section presents the optimized force field parameters for $[EMTr]^+$ and $[OBz]^-$ in anhydrous and aqueous [EMTr][OAc], [EMIm][OBz], and [EMTr][OBz], comparing them to the adapted literature force field.[1–3]

Table S1 presents the non-bonded interactions while the bonded interactions are shown in Table S2-S4. The force constants *(see Tab. S2 and S3)* and torsion parameters *(see Tab. S4)* do not include the factor 1/2. The 1–2 and 1–3 interactions were not taken into account while the 1–4 interactions were scaled with f_{ij}= 0.5. The Lennard–Jones interactions were calculated using geometric mixing rules. The PPPM long-range Coulomb solver implemented in LAMMPS[4] was used for Coulomb interactions. The coulomb and Lennard-Jones interactions were truncated at a cutoff radius of 800 pm. The force field BILFF[5] was applied for $[EMIm]^+$ and $[OAc]^-$ while for water, TIP4P–EW[6] (with fixed bonds and angles) was used without any modifications. The ions were assigned a total charge of ±0.82.

To optimize the force field parameters for $[EMTr]^+$, we utilized BILFF[5,7] as a starting point. The Lennard-Jones parameters and force constants for $[OBz]^-$ were adapted from OPLS–AA,[1–3] while the partial charges were obtained using the restrained electrostatic potential (RESP) methodology based on quantum chemical calculations. Furthermore, the reference bond length was determined by geometry optimization. For $[EMTr]^+$ the Lennard-Jones parameter were adapted of $[EMIm]^+$ from BILFF,[5] while the reference bond length and angle of atom type NR was determined by a geometry optimization. The used starting force field parameters are listed in Tab. S–2-5 in comparison to the new optimized parameters.

Table S1: Comparison of the atomic partial charges q and Lennard-Jones parameter σ and ϵ of $[EMTr]^+$ and $[OBz]^-$ in BILFF and the adapted literature force field.[1–3,5,8–10]

Atom Type	BILFF			Literature (adapted)[1–3,5,8–10]		
	q / e	σ / Å	ϵ / kJ mol^{-1}	q / e	σ / Å	ϵ / kJ mol^{-1}
			$[EMTr]^+$			
C1	−0.187	3.34	0.276	−0.147	3.34	0.276
CE	−0.054	3.34	0.276	−0.042	3.34	0.276
CW	−0.144	3.38	0.293	−0.133	3.38	0.293
HCW	0.191	1.48	0.126	0.150	1.54	0.126
HC	0.070	2.38	0.126	0.055	2.38	0.126
H1	0.148	2.38	0.126	0.116	2.38	0.126
NR	−0.204	3.10	0.711	−0.160	3.10	0.711
NA	0.204	3.10	0.711	0.160	3.10	0.711
			$[OBz]^-$			
C1	0.005	3.70	0.2929	0.006	3.55	0.2929
C2	−0.118	3.70	0.2929	−0.142	3.55	0.2929
C3	−0.121	3.70	0.2929	−0.025	3.55	0.2929
C4	−0.299	3.70	0.2929	−0.241	3.55	0.2929
CO	0.398	3.90	0.4393	0.371	3.75	0.4393
H2	0.070	2.42	0.1255	0.084	2.42	0.1255
H3	0.157	2.42	0.1255	0.069	2.42	0.1255
H4	0.200	2.42	0.1255	0.120	2.42	0.1255
O2	−0.550	2.80	0.8786	−0.524	2.96	0.8786

Table S2: Comparison of the bond equilibrium lengths l_0 and force constants k_l of $[EMTr]^+$ and $[OBz]^-$ in BILFF and the adapted literature force field.[1–3,5,8–10]

Bond	BILFF		Literature (adapted)[1–3,5,8–10]	
	l_0 / Å	k_l / kJ mol^{-1} Å^{-2}	l_0 / Å	k_l / kJ mol^{-1} Å^{-2}
		$[EMTr]^+$		
NA–NR	1.344	3199.2	1.340	3992.0
CW–HA	1.088	2633.8	1.085	2943.0
CW–NA	1.375	3108.7	1.403	2775.0
CW–CW	1.386	3773.2	1.374	4019.0
NA–CT	1.488	2046.3	1.485	2078.0
HC–CT	1.099	2679.4	1.099	3013.0
CT–CT	1.533	2125.5	1.532	2097.0
		$[OBz]^-$		
CA–CA	1.387	3274.1	1.400	1962.3
CA–HA	1.088	2707.4	1.084	1535.5
CA–CO	1.504	1906.9	1.529	1673.6
CO–O2	1.282	4273.1	1.252	2744.7

Table S3: Comparison of the angle equilibrium values θ_0 and force constants k_θ of $[EMTr]^+$ and $[OBz]^-$ in BILFF and the adapted literature force field.[1–3,5,8–10]

Angle	BILFF		Literature (adapted)[1–3,5,8–10]	
	θ_0 / Deg	k_θ / kJ mol^{-1} rad^{-2}	θ_0 / Deg	k_θ / kJ mol^{-1} rad^{-2}
		$[EMTr]^+$		
CW–NA–NR	112.1	568.7	112.1	585.8
NR–NA–CT	118.6	396.5	118.6	585.8
NA–NR–NA	104.4	610.1	104.4	585.8
NA–CT–CT	110.9	361.2	110.9	532.7
NA–CW–CW	107.0	579.7	107.0	805.7
NA–CW–HA	120.8	200.9	120.8	263.0
CW–CW–HA	131.7	190.5	131.7	236.7
NA–CT–HC	107.2	375.9	107.2	411.7
CT–CT–HC	111.4	296.2	111.4	338.6
HC–CT–HC	109.2	226.5	109.2	308.0
CW–NA–CT	125.2	242.9	125.2	367.8
		$[OBz]^-$		
CA–CA–CA	120.0	446.0	120.0	263.6
CA–CA–HA	120.0	258.1	120.0	146.4
CA–CA–CO	120.0	397.6	120.0	355.6
CA–CO–O2	117.0	550.2	117.0	292.9
O2–CO–O2	126.0	735.9	126.0	334.7

Table S4: Comparison of the torsional coefficients V_n of $[EMTr]^+$ and $[OBz]^-$ in BILFF and the adapted literature force field.[1–3,5,8–10]

Torsion Angle	BILFF V_1 / kJ mol^{-1}	BILFF V_2 / kJ mol^{-1}	BILFF V_3 / kJ mol^{-1}	BILFF V_4 / kJ mol^{-1}	Literature (adapted)[1–3,5,8–10] V_1 / kJ mol^{-1}	Literature (adapted) V_2 / kJ mol^{-1}	Literature (adapted) V_3 / kJ mol^{-1}	Literature (adapted) V_4 / kJ mol^{-1}
				$[EMTr]^+$				
CW–NA–NR–NA	0.0000	19.4600	0.0000	0.0000	0.0000	19.4600	0.0000	0.0000
CT–NA–NR–NA	0.0000	19.4600	0.0000	0.0000	0.0000	19.4600	0.0000	0.0000
NR–NA–CW–CW	0.0000	12.5500	0.0000	0.0000	0.0000	12.5500	0.0000	0.0000
NR–NA–CW–HA	0.0000	12.5500	0.0000	0.0000	0.0000	12.5500	0.0000	0.0000
NR–NA–CT–HC	0.0000	0.0000	0.0000	0.0000	0.0000	0.0000	0.0000	0.0000
NR–NA–CT–CT	0.1000	1.0000	0.1000	−0.3000	−5.2691	0.0000	0.0000	0.0000
CT–NA–CW–CW	0.0000	12.5500	0.0000	0.0000	0.0000	12.5500	0.0000	0.0000
CT–NA–CW–HA	0.0000	12.5500	0.0000	0.0000	0.0000	12.5500	0.0000	0.0000
NA–CW–CW–NA	0.0000	65.0000	0.0000	0.0000	0.0000	65.0000	0.0000	0.0000
NA–CW–CW–HA	0.0000	44.9800	0.0000	0.0000	0.0000	44.9800	0.0000	0.0000
HA–CW–CW–HA	0.0000	30.0000	0.0000	0.0000	0.0000	30.0000	0.0000	0.0000
CW–NA–CT–HC	0.1000	0.2000	0.0000	0.0000	0.0000	0.0000	0.5190	0.0000
CW–NA–CT–CT	0.4000	1.0000	0.0000	0.2000	14.3000	−12.2000	−1.5900	0.0000
NA–CT–CT–HC	0.0000	0.0000	0.3670	0.0000	0.0000	0.0000	0.3670	0.0000
HC–CT–CT–HC	0.0000	0.0000	1.2552	0.0000	0.0000	0.0000	1.2552	0.0000
				$[OBz]^-$				
CA–CA–CA–CA	0.0000	30.334	0.0000	0.0000	0.0000	30.334	0.0000	0.0000
HA–CA–CA–CA	0.0000	30.334	0.0000	0.0000	0.0000	30.334	0.0000	0.0000
HA–CA–CA–CO	0.0000	30.334	0.0000	0.0000	0.0000	30.334	0.0000	0.0000
HA–CA–CA–HA	0.0000	30.334	0.0000	0.0000	0.0000	30.334	0.0000	0.0000
CA–CA–CA–CO	0.0000	30.334	0.0000	0.0000	0.0000	30.334	0.0000	0.0000
CA–CA–CO–O2	0.0000	8.000	0.0000	0.0000	0.0000	8.000	0.0000	0.0000

2 Additional Radial Distribution Functions

To further characterize the force field and describe the underlying molecular systems, additional radial distribution functions (RDFs) are shown below.

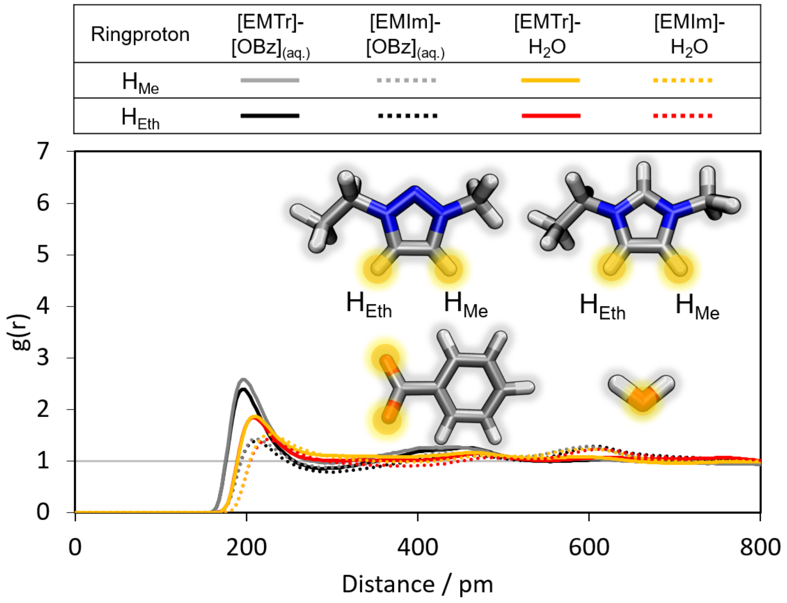

Figure S1: Comparison of the RDFs of the hydrogen bond between the marked oxygen atoms of $[OBz]^-$/water and the ring protons of $[EMIm]^+$/$[EMTr]^+$ calculated from force field MD simulations of [EMTr][OBz] and [EMIm][OBz] using BILFF.

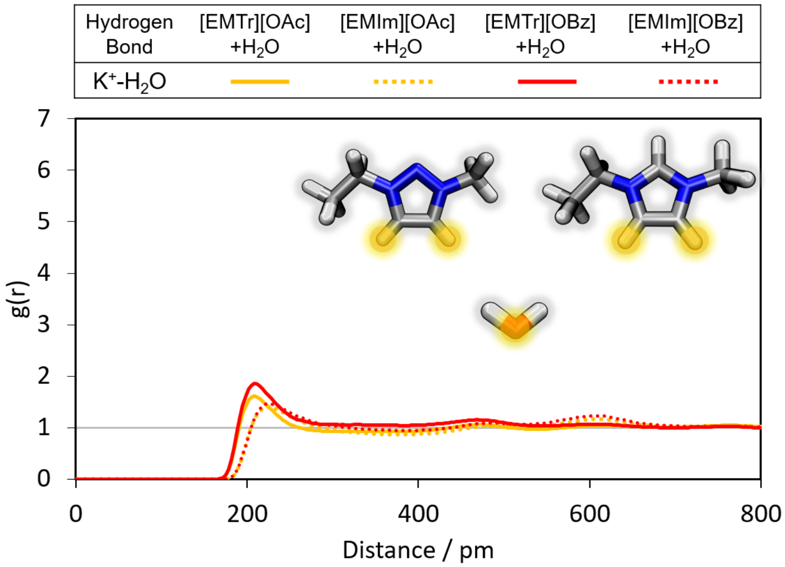

Figure S2: Comparison of the RDFs of the hydrogen bond between the marked oxygen atom of water and the ring protons of $[EMTr]^+$ and $[EMIm]^+$ calculated from force field MD simulations using BILFF in all four ILs.

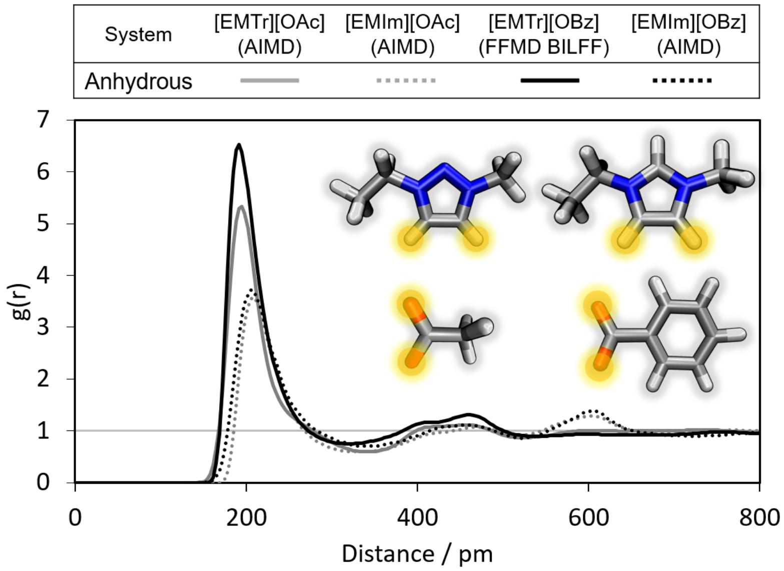

Figure S3: Comparison of the RDFs of the hydrogen bond between the marked oxygen atoms of $[OBz]^-$ as well as $[OAc]^-$ and the ring protons of $[EMIm]^+$ and $[EMTr]^+$ calulated from the reference AIMDs. For a comparison of the results with anhydrous [EMTr][OBz] the results from the force field MD simulation (FFMD) using BILFF are shown. The RDFs are averaged over the marked ring protons.

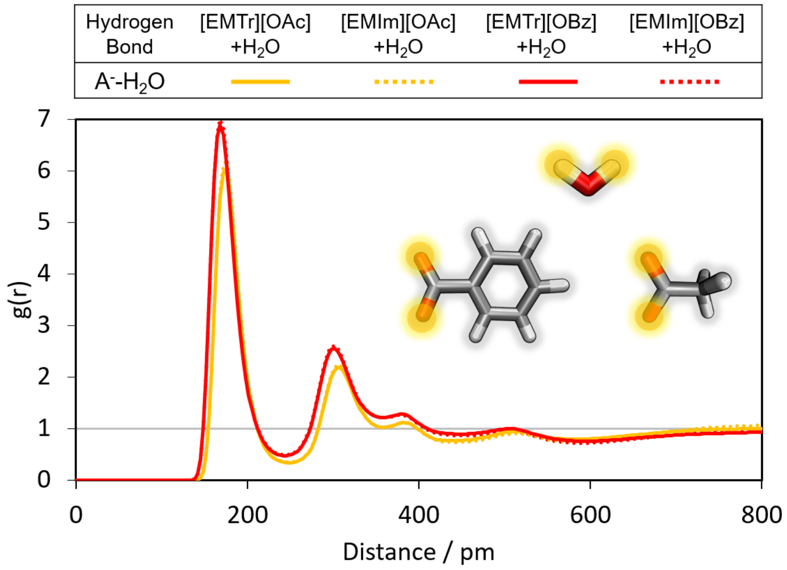

Figure S4: Comparison of the RDFs of the hydrogen bond between the marked oxygen atoms of $[OBz]^-$/$[OAc]^-$and the protons of water calculated from force field MD simulations using BILFF in all four ILs.

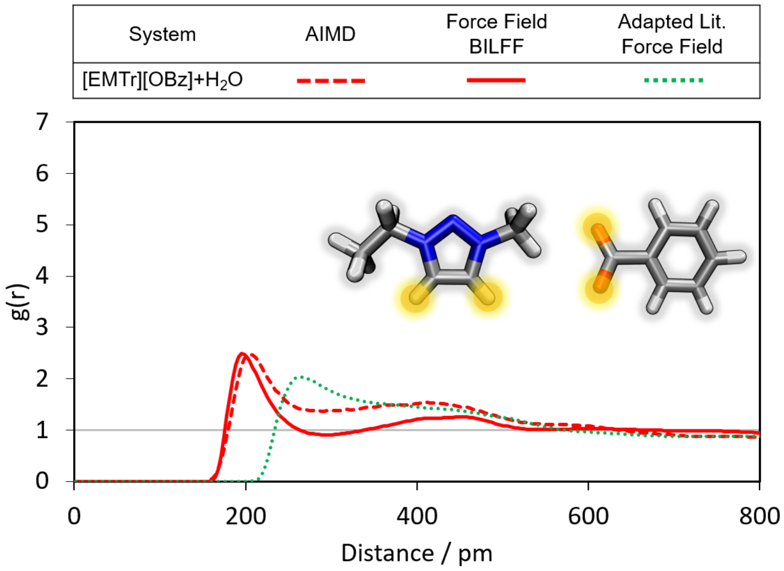

Figure S5: Comparison of the RDFs of the hydrogen bond between the marked oxygen atoms of $[OBz]^-$and the ring protons of $[EMTr]^+$ calculated from a reference AIMD and force field MD simulations using adapted literature force field parameter[8–10] and BILFF. The RDFs are averaged over both ring protons.

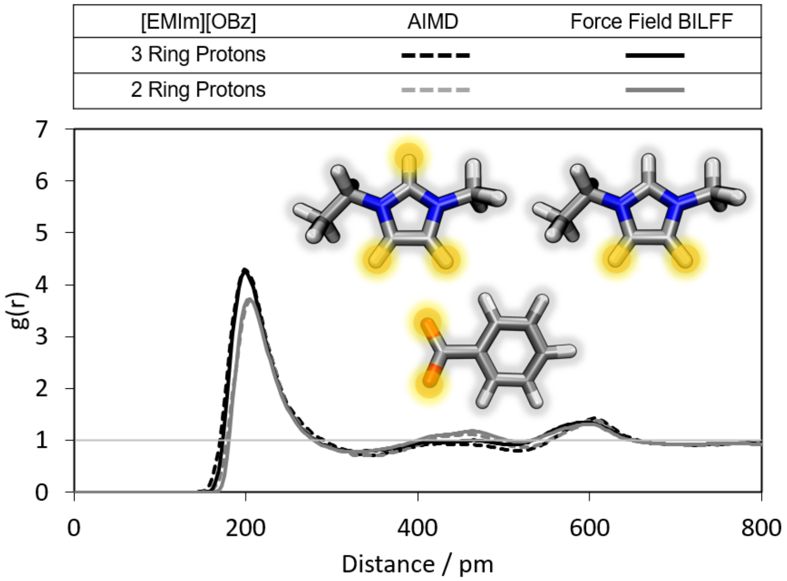

Figure S6: Comparison of the RDFs of the hydrogen bond between the marked oxygen atoms of $[OBz]^-$ and the two/three ring protons of $[EMIm]^+$ calculated from a reference AIMD and force field MD simulations using BILFF. The RDFs are averaged over the marked ring protons.

3 Additional Distance–Angle Combined Distribution Functions

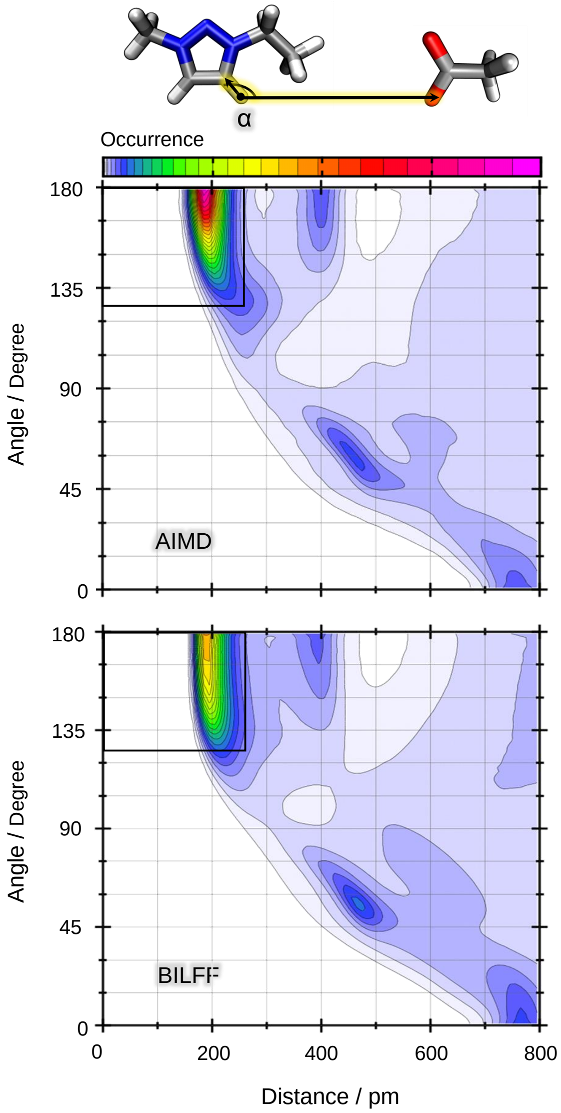

Figure S7: Distance–angle distribution function between an example ring proton of $[EMTr]^+$ and the oxygen atoms of $[OAc]^-$ in pure [EMTr][OAc] as a result of a reference AIMD (top) and a force field MD simulations with our new force field (bottom). The black rectangle demonstrates the geometric criterion for calculating the lifetime of the hydrogen bonds.

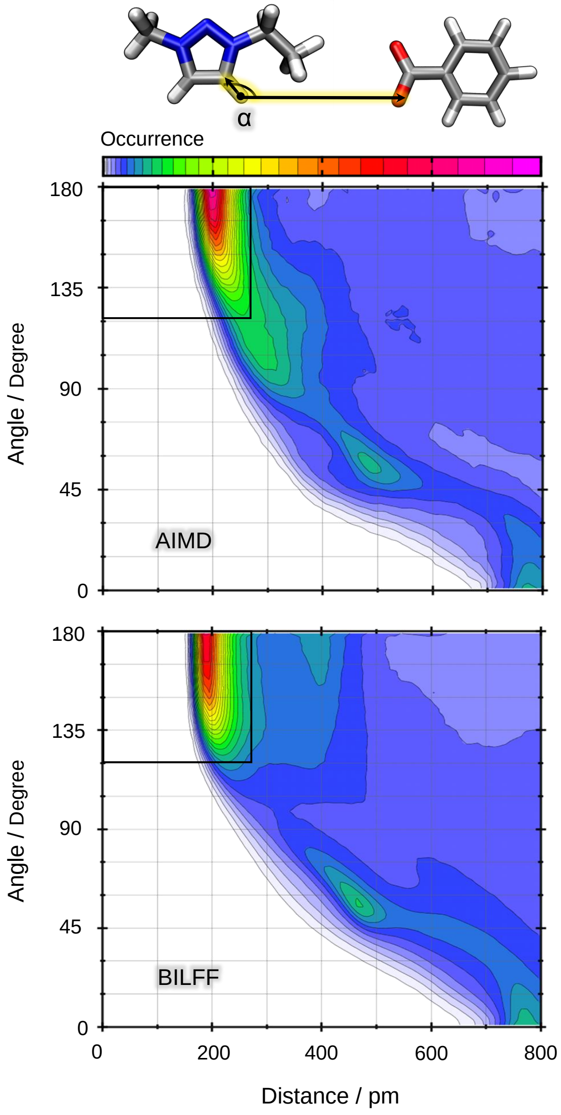

Figure S8: Distance–angle distribution function between an example ring proton of $[EMTr]^+$ and the oxygen atoms of $[OBz]^-$ in aqueous [EMTr][OBz] as a result of a reference AIMD (top) and a force field MD simulations with our new force field (bottom). The black rectangle demonstrates the geometric criterion for calculating the lifetime of the hydrogen bonds.

4 Additional Spatial Distribution Function

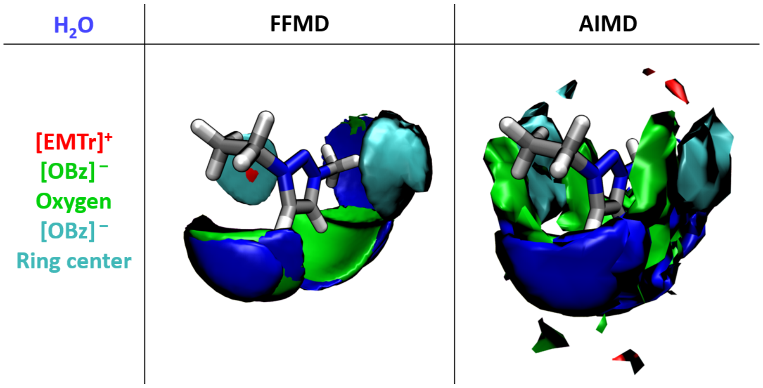

Figure S9: Spatial distribution function of the arrangement of molecules in aqueous [EMTr][OBz] around the cation with the protons and oxygen atoms of water (blue, 26 nm^{-3}) and the oxygen atom of the anion (green, 7 nm^{-3}) as well as the ring center of the cation (red, 7 nm^{-3}) and anion (cyan, 10 nm^{-3}) resulted from a force field MD simulation using BILFF and the reference AIMD simulation.

5 Sankey Diagrams

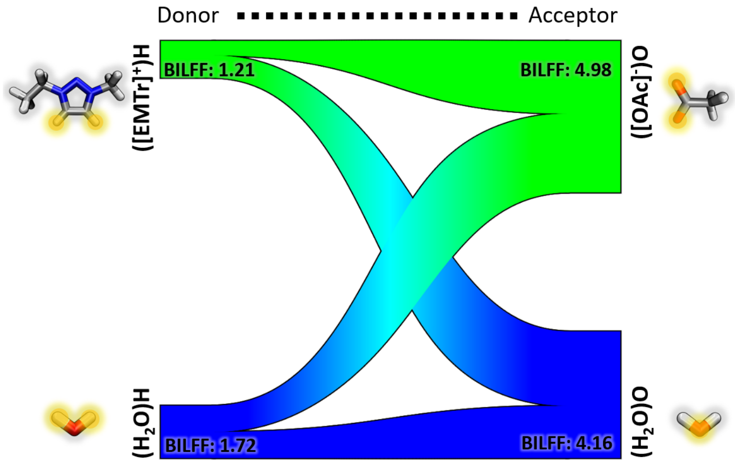

Figure S10: Sankey diagram of aqueous [EMTr][OAc] calculated from a force field MD simulation using BILFF.

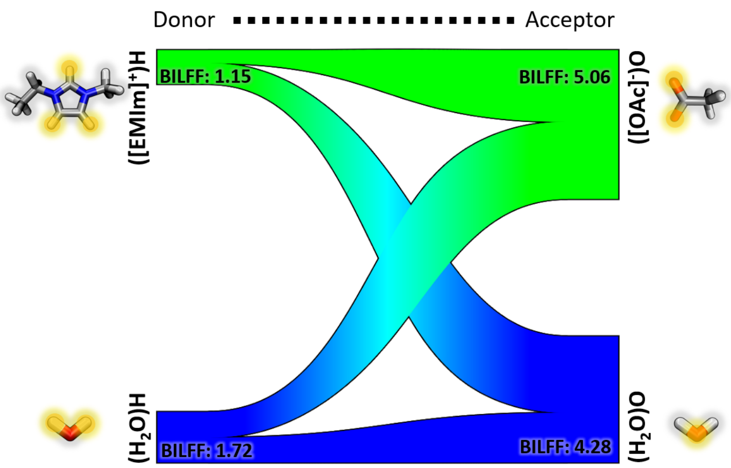

Figure S11: Sankey diagram of aqueous [EMIm][OAc] calculated from a force field MD simulation using BILFF.

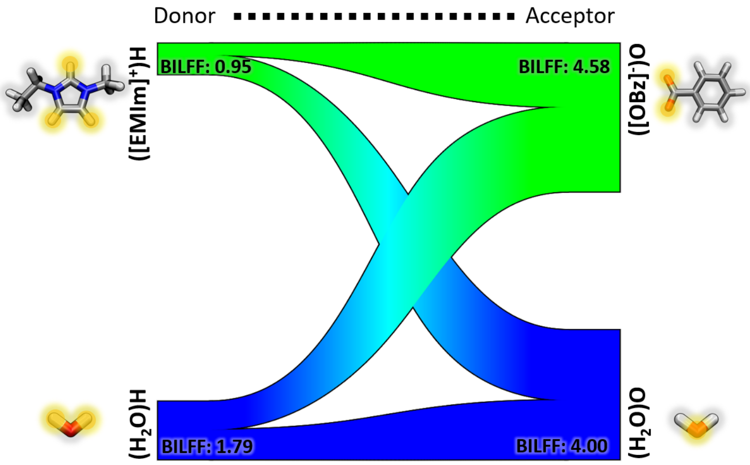

Figure S12: Sankey diagram of aqueous [EMIm][OBz] calculated from a force field MD simulation using BILFF.

6 Lifetime of Additional Hydrogen Bonds

Table S5: Angle and distance criteria of the different hydrogen bonds for the calculation of the hydrogen bond lifetime.

Hydrogen Bond	Atom Distance / pm	Angle / °
([EMTr])H–O([OAc])	0–260	125–180
([EMIm])H–O([OBz])	0–250	125–180
([EMTr])H–O([OBz])	0–270	122–180
([EMTr])H–O(H_2O)	0–260	118–180
(H_2O)H–O([OBz])	0–230	137–180
([EMIm])H–O([OAc])	0–260	112–180
([EMIm])H–O(H_2O)	0–260	120–180
(H_2O)H–O([OAc])	0–180	165–180

Table S6: Overview of the lifetime τ of the hydrogen bonds in all four systems comparing the results of the reference AIMD simulation and the force field MD simulation using BILFF at the given temperatures (C^+=Cation, A^-=Anion). (No AIMD simulations of anhydrous [EMTr][OBz] as well as [EMTr][OAc]/H_2O and [EMIm][OBz]/H_2O have been calculated, so no data are available for these.)

Temp. / K	Intermittent τ(AIMD) / ps	Intermittent τ(FFMD) / ps	Continuous τ(AIMD) / ps	Continuous τ(FFMD) / ps
		[EMTr][OAc]		
$(C^+)H_{Eth}\cdots O(A^-)$				
350 K	513.8	556.6	4.1	4.3
$(C^+)H_{Me}\cdots O(A^-)$				
350 K	581.6	394.5	3.8	3.9
		[EMTr][OAc]/H_2O		
$(C^+)H_{Eth}\cdots O(A^-)$				
350 K	–	74.9	–	1.9
$(C^+)H_{Me}\cdots O(A^-)$				
350 K	–	81.9	–	1.8
$(C^+)H_{Eth}\cdots O(H_2O)$				
350 K	–	24.9	–	1.1
$(C^+)H_{Me}\cdots O(H_2O)$				
350 K	–	23.9	–	1.0
		[EMIm][OAc] [a]		
$(C^+)H_{Eth}\cdots O(A^-)$				
350 K	212.6	250.3	10.5	10.0
$(C^+)H_{Me}\cdots O(A^-)$				
350 K	282.4	260.7	11.9	11.5
		[EMIm][OBz]		
$(C^+)H_{Eth}\cdots O(A^-)$				
350 K	309.6	750.1	1.1	1.5
$(C^+)H_{Me}\cdots O(A^-)$				
350 K	161.7	657.3	1.0	1.4

		[EMIm][OBz]/H_2O		
$(C^+)H_{Eth} \cdots O(A^-)$				
350 K	–	71.8	–	0.9
$(C^+)H_{Me} \cdots O(A^-)$				
350 K	–	69.1	–	0.8
$(C^+)H_{Eth} \cdots O(H_2O)$				
350 K	–	25.2	–	0.6
$(C^+)H_{Me} \cdots O(H_2O)$				
350 K	–	24.8	–	0.6
		[EMTr][OBz]		
$(C^+)H_{Eth} \cdots O(A^-)$				
350 K	–	1712.7	–	6.7
$(C^+)H_{Me} \cdots O(A^-)$				
350 K	–	2014.9	–	5.8
		[EMTr][OBz]/H_2O		
$(C^+)H_{Eth} \cdots O(A^-)$				
350 K	80.7	128.2	1.8	2.5
450 K	25.8	18.3	0.9	1.4
550 K	10.9	7.6	0.6	1.0
$(C^+)H_{Me} \cdots O(A^-)$				
350 K	83.8	134.5	1.4	2.4
450 K	17.9	9.2	0.7	1.3
550 K	10.8	7.8	0.6	1.0
$(C^+)H_{Eth} \cdots O(H_2O)$				
350 K	38.9	33.0	0.7	1.1
450 K	4.4	4.6	0.4	0.7
550 K	–	1.7	0.3	0.5
$(C^+)H_{Me} \cdots O(H_2O)$				
350 K	22.7	33.0	0.6	1.0
450 K	4.7	4.6	0.3	0.6
550 K	–	1.8	0.3	0.5

[a] *Calculated from the MD simulations of our already published article.* [5]

References

[1] W. L. Jorgensen, D. S. Maxwell and J. Tirado-Rives, J. Am. Chem. Soc., 1996, **118**, 11225–11236.

[2] J. W. Ponder and D. A. Case, Adv. Protein Chem., 2003, **66**, 27–85.

[3] S. V. Sambasivarao and O. Acevedo, J. Chem. Theory Comput., 2009, **5**, 1038–1050.

[4] S. Plimpton, J. Comp. Phys., 1995, **117**, 1–19.

[5] E. Roos and M. Brehm, PCCP, 2021, **23**, 1242–1253.

[6] H. W. Horn, W. C. Swope, J. W. Pitera, J. D. Madura, T. J. Dick, G. L. Hura and T. Head-Gordon, J. Chem. Phys., 2004, **120**, 9665–9678.

[7] E. Roos, D. Sebastiani and M. Brehm, PCCP, 2023, **25**, 8755–8766.

[8] J. N. Canongia Lopes, J. Deschamps and A. A. H. Pádua, J. Phys. Chem. B, 2004, **108**, 2038–2047.

[9] J. N. Canongia Lopes and A. A. H. Pádua, J. Phys. Chem. B, 2006, **110**, 19586–19592.

[10] J. N. Canongia Lopes and A. A. H. Pádua, Theor. Chem. Acc., 2012, **131**, 3330.
